# A dominant mutation in *β-AMYLASE1* disrupts nighttime control of starch degradation in Arabidopsis leaves

**DOI:** 10.1093/plphys/kiab603

**Published:** 2021-12-27

**Authors:** Doreen Feike, Marilyn Pike, Libero Gurrieri, Alexander Graf, Alison M Smith

**Affiliations:** John Innes Centre, Norwich Research Park, Norwich NR4 7UH, UK; John Innes Centre, Norwich Research Park, Norwich NR4 7UH, UK; Department of Pharmacy and Biotechnology, University of Bologna, Bologna 40126, Italy; John Innes Centre, Norwich Research Park, Norwich NR4 7UH, UK; John Innes Centre, Norwich Research Park, Norwich NR4 7UH, UK

## Abstract

Arabidopsis (*Arabidopsis thaliana*) leaves possess a mechanism that couples the rate of nighttime starch degradation to the anticipated time of dawn, thus preventing premature exhaustion of starch and nighttime starvation. To shed light on the mechanism, we screened a mutagenized population of a starvation reporter line and isolated a mutant that starved prior to dawn. The mutant had accelerated starch degradation, and the rate was not adjusted to time of dawn. The mutation responsible led to a single amino acid change (S132N) in the starch degradation enzyme BETA-AMYLASE1 (BAM1; mutant allele named *bam1-2D*), resulting in a dominant, gain-of-function phenotype. Complete loss of BAM1 (in *bam1-1*) did not affect rates of starch degradation, while expression of BAM1(S132N) in *bam1-1* recapitulated the accelerated starch degradation phenotype of *bam1-2D*. In vitro analysis of recombinant BAM1 and BAM1(S132N) proteins revealed no differences in kinetic or stability properties, but in leaf extracts, BAM1(S132N) apparently had a higher affinity than BAM1 for an established binding partner required for normal rates of starch degradation, LIKE SEX FOUR1 (LSF1). Genetic approaches showed that BAM1(S132N) itself is likely responsible for accelerated starch degradation in *bam1-2D* and that this activity requires LSF1. Analysis of plants expressing BAM1 with alanine or aspartate rather than serine at position 132 indicated that the gain-of-function phenotype is not related to phosphorylation status at this position. Our results strengthen the view that control of starch degradation in wild-type plants involves dynamic physical interactions of degradative enzymes and related proteins with a central role for complexes containing LSF1.

## Teaser

A single amino acid change in Arabidopsis BETA-AMYLASE1 prevents coupling of night-time starch degradation to time of dawn and causes premature exhaustion of starch reserves in the dark.

## Introduction

The leaves of many plants produce starch as well as sucrose as primary products of photosynthesis during the day. Sucrose is used for growth and metabolism in the source leaf or in sink organs of the plant following its export via the phloem, whereas starch accumulates inside chloroplasts as insoluble, semi-crystalline granules made of α1,4, α1,6-linked glucose polymers. During the night, hydrolysis of the accumulated starch and conversion of the resulting maltose and glucose to sucrose in the cytosol ensures a continuing supply of sucrose for growth and metabolism in the absence of photosynthesis. Remarkably, the rate of starch mobilization in Arabidopsis (*Arabidopsis thaliana*) leaves during the night is essentially linear and is such that the starch reserve is almost but not completely exhausted by dawn. This pattern of mobilization results in a continuous and relatively constant supply of sucrose across the day–night cycle, so preventing carbon starvation and making efficient use within each 24-h period of carbon gained during the light period (Smith and Stitt, 2007; Smith and Zeeman, 2020).

These features of starch mobilization during the night are remarkably robust across a wide range of conditions. Degradation is linear and starch lasts until dawn during nights between 12- and 20-h long, and also following unanticipated changes in the time of onset of darkness (an “early night”). We showed previously that the plant circadian clock is centrally involved in the adjustment of the rate of degradation at night. Evidence included data from clock mutants that anticipate days of ˂24 h: starch in these mutants was completely degraded before dawn and plants exhibited starvation in the last few hours of the night. Modeling of the control of degradation revealed that the mechanism must use information about both time remaining until dawn and starch content in leaf cells and predict the properties of hypothetical components of the mechanism (Graf et al., 2010; Scialdone et al., 2013).

To discover the components of the mechanism, we conducted a screen to identify mutant plants unable to exercise appropriate control over the rate of starch degradation at night (Feike et al., 2016). Mutants were generated in a line of plants containing a luciferase gene on an Arabidopsis “starvation” promoter. The promoter is active only when sugars fall to very low levels. Luciferase is not expressed during the normal day–night cycle in this line, but is expressed and can be detected as luminescence in an extended night in which darkness continues beyond the normal dawn. We mutagenized this reporter line and looked for mutant plants that displayed luminescence before the end of a normal night, indicative of premature starvation. Identification of the mutated genes could shed light on the mechanism that controls starch degradation.

The utility of this starvation screen is illustrated by our discovery of a previously unknown class of proteins essential for normal starch degradation. A mutant plant exhibiting premature starvation was found to have a greatly accelerated rate of starch degradation at night because of loss of a protein termed EARLY STARVATION1 (ESV1; At1g42430; Feike et al., 2016). This protein and a related protein LIKE ESV (At3g55760) are conserved in green plants and are found in the chloroplast stroma and bound to starch granules. Genetic analysis and examination of starch granules led us to suggest that ESV1 acts upstream of the pathway of starch degradation, and may influence the accessibility of the starch granules to starch-degrading enzymes (Feike et al., 2016). The precise function of the protein is not yet known.

Here we show that a second mutant from the starvation screen also has accelerated starch degradation at night, and as a consequence consumes all of its starch reserves and starves before dawn. The mutation causes a single amino acid change in the gene encoding a β-amylase, BETA-AMYLASE1 (BAM1; At3g23920), an isoform located in plastids and capable of starch degradation in vivo (Sparla et al., 2006; Horrer et al., 2016; Thalmann et al., 2016; Zanella et al., 2016). The acceleration of starch degradation caused by the mutation is dominant: accordingly, we named the mutant allele *bam1-2D*. This is a most surprising result because complete loss of BAM1 (in the *bam1-1* mutant) does not alter the rate of starch degradation in mesophyll chloroplasts in a wild-type background (Fulton et al., 2008). We explore the biochemical and genetic consequences of the *bam1-2D* mutation, and conclude that BAM1 is part of a chloroplastic protein complex that is essential for the normal control of starch degradation.

## Results

### Identification and characterization of a mutation that prevents normal control of nighttime starch degradation

**Figure 1:**
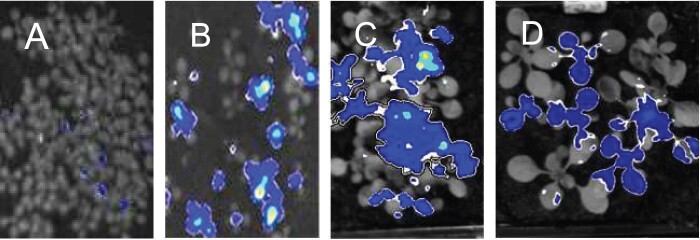
Evidence for a dominant mutation. Plants grown with 12-h light, 12-h dark were subjected to an early night (starting 8-h after dawn) and analyzed for luminescence at the end of the night. A, Seedlings of the parental reporter line. B and C, Seedlings of the M3 and M4 generations, respectively, of the mutant. D, Plants of the F1 generation of a cross between the mutant and *Ler*. About half of the plants are luminescent.

Mutagenized plants (M2 generation, 10 d old, growth conditions of 12-h light, 12-h dark) carrying luciferase expressed from the promoter of the starvation gene At1g10070 (Graf et al., 2010; Feike et al., 2016) were screened for luminescence just before dawn at the end of either a normal night (12-h darkness) or a night in which darkness had been imposed at 8 h after dawn rather than the standard 12 h (referred to as an early night, 16-h darkness). As expected, unmutagenized plants were not luminescent at either point (Feike et al. 2016). We identified, and confirmed in the M3 generation, a mutant that exhibited low-level luminescence at the end of a normal night and strong luminescence at the end of an early night. Genetic analysis of the mutant revealed that the mutation was dominant (Figure  1). The progeny from M3 and M4 plants segregated for plants with strong luminescence at the end of an early night and plants with no luminescence at this point. In the F1 generation of crosses between M3 plants and either the parental reporter line or Landsberg *erecta* (L*er*), about half of the plants displayed strong luminescence at the end of an early night. Neither the predominance of luminescent plants in the segregating M3 and M4 generations nor the presence of luminescent plants in F1 progeny would be expected if the mutation were recessive.

The underlying mutation was identified by map-based cloning. The mutant (Columbia-0 [Col-0] background) was crossed to a wild-type L*er* plant and F_2_ plants were screened for luminescence at the end of the early night. Because the mutation is dominant homozygous mutants could not be identified by phenotype, so DNA was extracted from F_2_ plants that retained the reporter gene but had no luminescence at the end of an early night: these plants will be homozygous for the wild-type allele from the L*er* parent.

Mapping located the mutation in an interval of 1.4 Mb spanning 453 genes on chromosome 3. The region contained a locus known to encode an enzyme of starch metabolism: At3g23920, which encodes BAM1 (Sparla et al., 2006; Fulton et al., 2008). Sequencing revealed a G/A transition mutation in the first exon of this gene that changes codon AGT to AAT, thus causing a serine to asparagine substitution at position 132 of the protein. This allele of *BAM1* was named *bam1-2D* (*bam1-1* is a loss-of-function T-DNA insertion mutant SALK_039895 described in Fulton et al., 2008), and the protein encoded by the allele is referred to as BAM1(S132N). As a check, we also sequenced the promoter region (1-kb upstream of the start codon) of the *BAM1* gene from wild-type and *bam1-2D* lines. This sequence was identical in the two genotypes.

**Figure 2:**
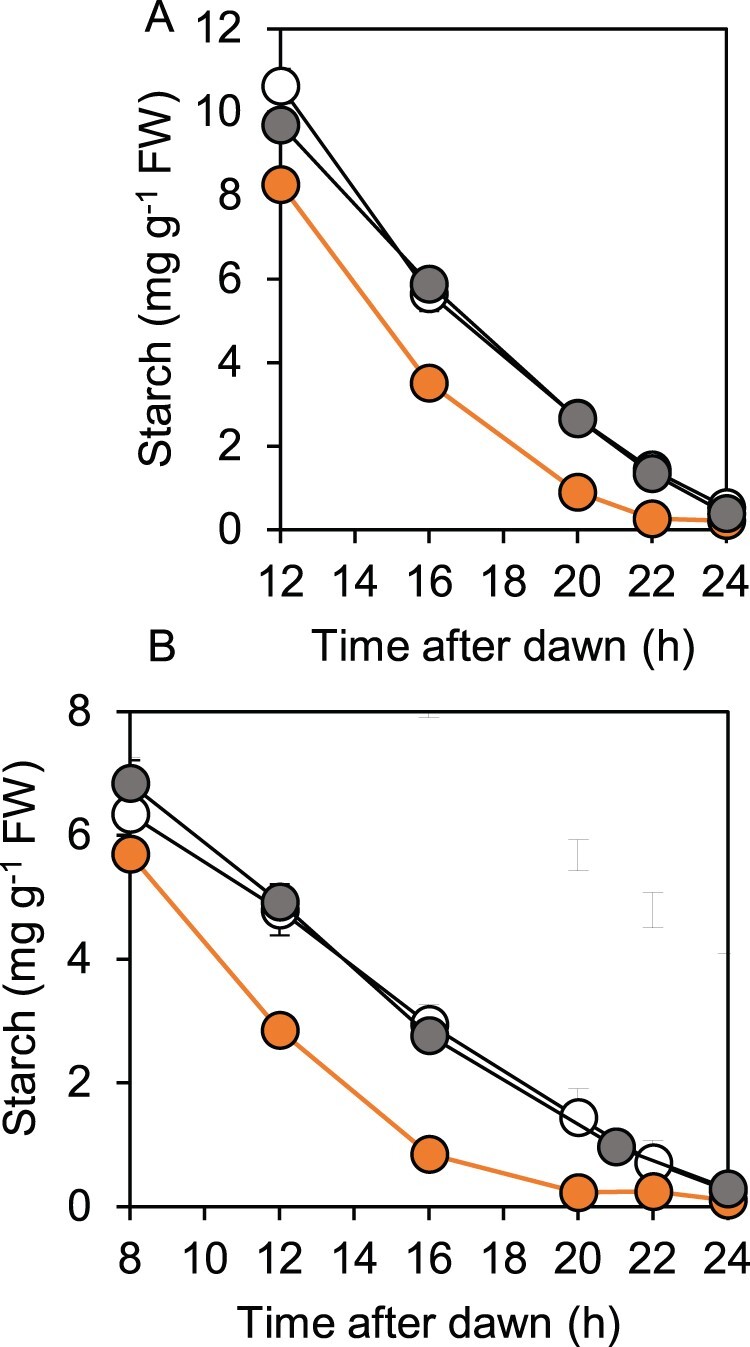
Patterns of starch degradation in wild-type, *bam1-1*, and *bam1-2D* plants. Plants were grown with 12-h light, 12-h dark for 21 d, then rosettes were harvested and their starch contents measured. A, Starch content during a normal 12-h night. B, Starch content during an early night, starting at 8 h after dawn. Data are means of measurements on five individual rosettes. Error bars are SE: where not visible, they are smaller than the symbol. Open symbol, wild-type. Gray symbol, *bam1-1*. Orange symbol, *bam1-2D*.

Consistent with the predawn luminescence phenotype of *bam1-2D*, starch reserves in this mutant were exhausted before dawn during both a normal 12-h night and an early night (Figure  2). In contrast, and as expected from our previous studies, starch reserves were not depleted until dawn during either a normal or an early night in wild-type and *bam1-1* plants (Fulton et al., 2008; Graf et al., 2010; Scialdone et al., 2013). When starch degradation rates during an early night were plotted as percentage of end-of-day starch (Supplemental Figure S1), it was apparent that while the rate of degradation in wild-type and *bam1-1* plants was slower in an early than in a normal night, the rate in *bam1-2D* plants was essentially the same in a normal and an early night. In other words, the rate of degradation was not adjusted to the length of the night in *bam1-2D* plants. Calculation of relative starch degradation rates in the first 8 h of an early night showed that *bam1-2D* plants degraded 11% of end-of-day starch per hour, whereas wild-type plants degraded only 5% of end-of-day starch per hour. In most experiments the starch content of *bam1-2D* plants at the end of the day was lower than that of wild-type plants. It seems likely that this reflects starch degradation at the same time as synthesis during the day: the same phenomenon occurs in *esv1*, another mutant with accelerated nighttime starch degradation (Feike et al., 2016).

**Figure 3:**
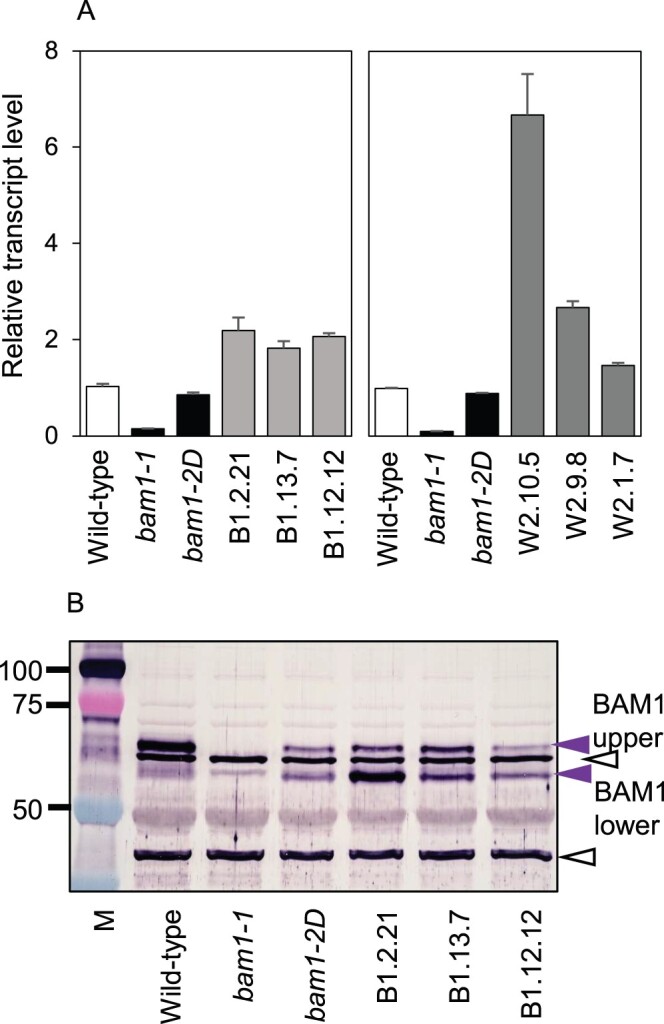
Transcript and protein abundance of BAM1 in wild-type, mutant and transgenic plants. A, Levels of *BAM1* transcript in rosettes (19 d old), measured by RT-qPCR and expressed relative to the level of *UBQ10* transcript. Left, *bam1-1*, *bam1-2D*, and three transgenic lines expressing *bam1-2D* in a *bam1-1* background. Right, *bam1-1*, *bam1-2D*, and three transgenic lines expressing *bam1-2D* in a wild-type background. Values are means ± standard error (se) of measurements made on three biological replicates. B, Immunoblot of a denaturing gel of extracts of the lines from (A). The blot was probed with the BAM1 antiserum described by Thalmann et al. (2016) at a concentration of 1/1,200. Open black arrows indicate bands recognized by the antiserum which are also present in the *bam1-1* null mutant and are therefore not BAM1. They serve as loading controls. Purple arrows indicate two bands attributable to BAM1. Masses of markers (M) are in kilodalton.

We checked whether the *bam1-2D* mutation affected transcript and protein levels (Figure  3). *BAM1* transcript was not detected in previously described *bam1-1* mutants, which have wild-type patterns of starch degradation in standard growth conditions (Fulton et al., 2008). Levels of *BAM1* transcript in the *bam1-2D* mutant were similar to those in wild-type plants. Immunoblotting (using a BAM1-specific antiserum: Thalmann et al., 2016) of gels of leaf extracts revealed two bands with appropriate masses that were present in wild-type leaves but not in *bam1-1* leaves (Figure  3B). Both bands are thus likely to be products of the *BAM1* gene (confirmed by peptide sequencing, described in the next section). The upper of the two bands (“BAM1 upper” in Figure  3B) was present in *bam1-2D* extracts but at substantially lower levels than in wild-type plants. The lower of the two bands (“BAM1 lower” in Figure  3B) was present at comparable levels in mutant and wild-type extracts. The total amount of BAM1 protein thus appeared to be lower in mutant than in wild-type leaves. We used a different type of gel and a different BAM1-specific antiserum to check this finding (Supplemental Figure S2). The results supported the view that BAM1 protein is substantially less abundant in *bam1-2D* than in wild-type leaves.

In vitro assays of recombinant BAM1 did not reveal any major differences in catalytic properties between mutant and wild-type proteins (Supplemental Figure S3). The predicted mature BAM1 and BAM1(S132N) proteins with C-terminal -his tags were expressed in *Escherichia coli*, the recombinant proteins were purified on nickel columns, and the -his tag was removed prior to assay. The activities of the two proteins were very similar with respect to specific activity, pH dependence, and thermostability. Incubation for 1 h at 37°C under oxidizing conditions reduced the activity of BAM1 by 84% and BAM1(S132N) by 81% relative to incubation under reducing conditions and incubation for 3 h at room temperature reduced the activity of both proteins by 46%.

In summary, the accelerated rates of starch degradation in *bam1-2D* mutants cannot be explained either by elevated levels of BAM1 protein or from in vitro assessments of the catalytic properties of the protein.

### Recapitulation of the *bam1-2* phenotype by expression of BAM1(S132N) in a *bam1* null mutant

To provide independent evidence about the involvement of the *bam1-2D* mutation in the accelerated rate of starch degradation, we expressed BAM1(S132N) from the 35S promoter in *bam1-1* mutant plants. If the *bam1-2D* mutation is responsible for the accelerated starch degradation, we expected that introduction of the mutant protein into *bam1-1* would result in accelerated rates of starch degradation in this null background.

Multiple transgenic lines expressing BAM1(S132N) were generated, and three transgenic lines with *bam1-2D* transcript levels approximately twice as high as *BAM1* transcript levels in wild-type plants were selected for further study (Figure  3A). Immunoblotting showed that, like *bam1-2D*, these transgenic lines had substantially lower levels of protein in the upper putative BAM1 band than wild-type plants. They appeared to have more protein in the putative BAM1 lower band than wild-type plants (Figure  3B). To check on the identity of the bands, upper and lower bands from sodium dodecyl sulfate-containing (SDS) gels of material enriched for BAM1 protein by immunoprecipitation from soluble extracts with BAM1 antiserum were separately excised and subjected to peptide sequencing. As expected, no peptides from BAM1 were detected in control samples from the *bam1-1* mutant. Peptides recovered from the upper band (obtained from wild-type extracts) gave 56% coverage of the predicted protein, and peptides recovered from the lower band (obtained from extracts of the transgenic line expressing BAM1(S132N)) gave a 69% coverage of the predicted protein (Supplemental Table S1). All of the peptides conformed to the expected amino acid sequence of the protein. These data confirm that both upper and lower bands are BAM1 proteins, and rule out the possibility that the lower band is a cleavage product of the upper band. None of the peptides recovered from any of the genotypes were from the region in which the S132N amino acid change is located.

**Figure 4:**
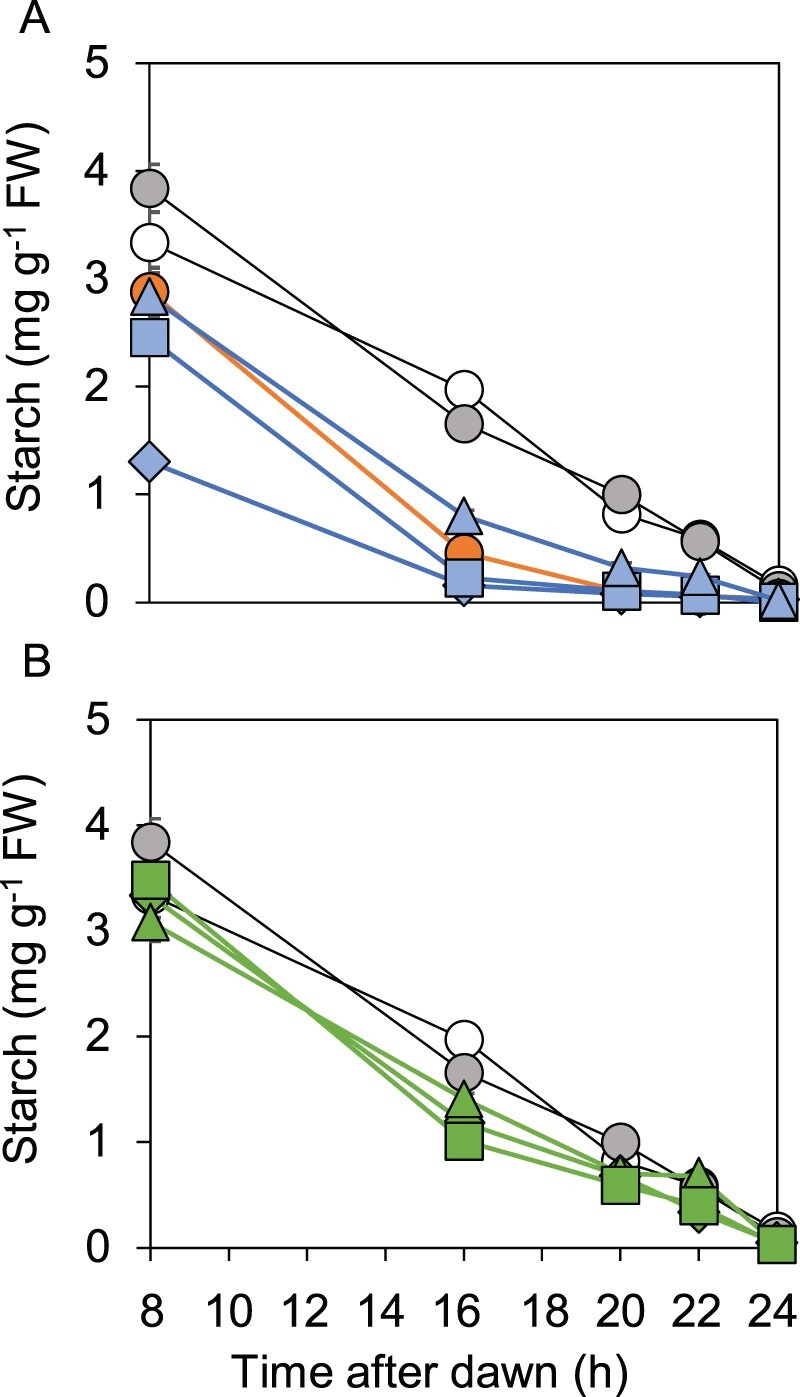
Comparison of patterns of starch degradation in wild-type, *bam1-1* and *bam1-2D* plants and transgenic lines. Plants were grown with 12-h light, 12-h dark for 21 d, then rosettes were harvested and starch contents were measured during an early night, which started 8-h after dawn. A, Open, gray and orange symbols represent, respectively, wild-type, *bam1-1* and *bam1-2D* plants. Blue symbols represent three independent lines of transgenic plants expressing BAM1(S132N) in a *bam1-1* background. Triangle, B1.12.12. Square, B1.13.7. Diamond, B1.2.21. B, Open and gray symbols represent, respectively, wild-type and *bam1-1* plants. Green symbols represent three independent lines of transgenic plants expressing BAM1(S132N) in a wild-type background. Triangles, W1.9.8. Squares, W1.1.7. Diamonds, W1.10.5. Data are means of measurements on five individual rosettes. Error bars are se: where not visible, they are smaller than the symbol.

We compared starch contents of wild-type, *bam1-1* and *bam1-2D* mutants and the three transgenic lines expressing BAM1(S132N) during an early night, a condition that resulted in a particularly pronounced difference in starch degradation profiles between wild-type and *bam1-2* mutant plants (see Figure  2). The results showed unambiguously that introduction of BAM1(S132N) into the *bam1-1* mutant recapitulated the accelerated rates of starch degradation seen in the *bam1-2D* mutant (Figure  4; Supplemental Figure S4; Supplemental Table S2). Starch reserves were exhausted by 12 h into the 16-h night in all three transgenic lines.

As a further check on the phenotypic consequences of expression of the *bam1-2D* allele, we introduced this gene into wild-type plants. Because the *bam1-2D* mutation is dominant in the sense that starch degradation is accelerated in plants carrying one mutant and one wild-type *BAM1* allele (Figure  1), it seemed possible that the resulting transgenic plants would have accelerated rates of starch degradation even though wild-type BAM1 protein is present. From multiple transgenic lines, we selected three that had between 1.5 and 7 times more *BAM1* transcript than wild-type plants (Figure  3A). Although none of lines exhausted their starch reserves prematurely during a normal or early night (Figure  4B; Supplemental Figure S4), for two of the three transgenic lines the rate of starch degradation in the first 8 h of an early night was statistically significantly higher than in wild-type plants (Supplemental Table S2). We suggest that the level of BAM1(S132N) activity may have been insufficient to have a major effect on starch degradation in plants with two wild-type *BAM1* alleles and hence a full complement of BAM1 protein.

### Like BAM1, BAM1(S132N) interacts with LSF1

Schreier et al. have recently shown that a fraction of the BAM1 protein in wild-type Arabidopsis chloroplasts exists in a complex with other proteins: they identified the proteins LIKE SEX FOUR1 (LSF1) and the chloroplastic NAD-malate dehydrogenase (pNAD-MDH) as interacting proteins (Schreier et al., 2018, 2019). It was proposed that LSF1—which possesses a starch-binding domain—may act as a scaffold that recruits other proteins to the starch granule surface. Although LSF1 is similar in sequence to proteins that catalyze the dephosphorylation of starch granules (STARCH EXCESS4 [SEX4] and LSF2 Kötting et al., 2009; Santelia et al., 2011), it lacks some amino acid residues essential for phosphatase activity and has no detectable enzymatic activity. Nonetheless, it is essential for normal starch degradation at night: mutants lacking LSF1 have the high end-of-day and end-of-night starch contents (a starch-excess phenotype) typical of mutants with defects in starch degradation (Comparot-Moss et al., 2010; Scialdone et al., 2013).

**Figure 5:**
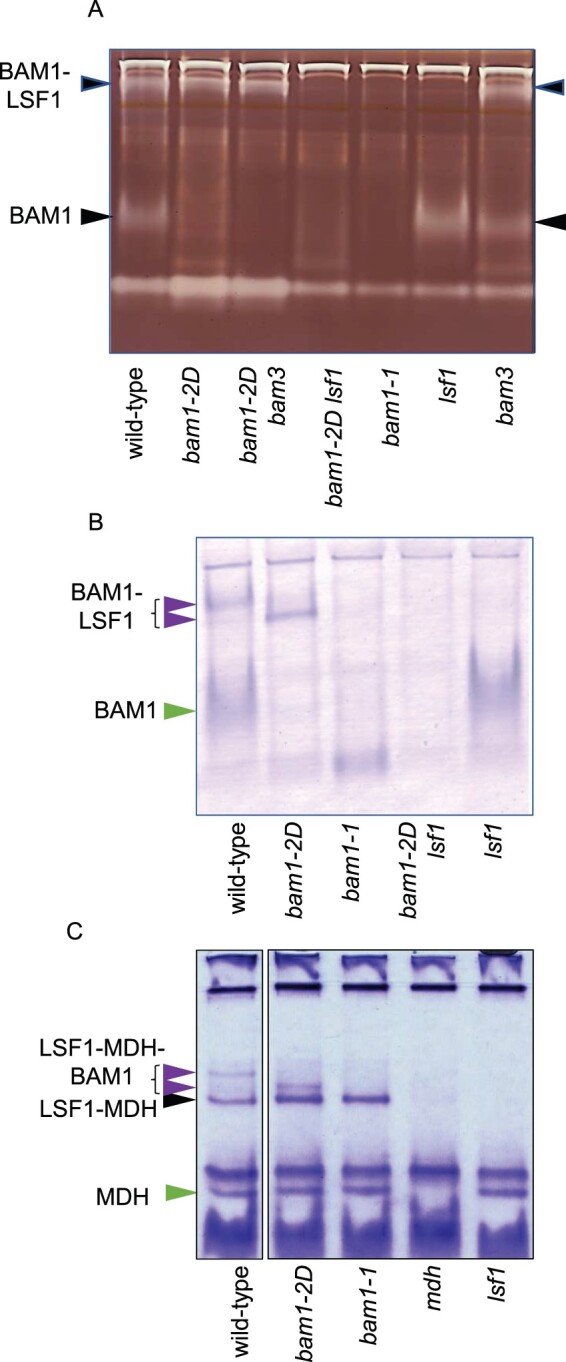
Native gels and immunoblots of extracts of wild-type, mutant, and double mutant plants. A, Native gel containing amylopectin, stained with iodine to reveal starch-degrading activities. The identity of arrowed bands is deduced from Schreier et al. (2019) and from the presence/absence of the bands in different mutants. B, Blot of a native gel without amylopectin, developed with the antiserum against BAM1 described by Fulton et al. (2008). Note that the position of the BAM1-LSF1 band is different for wild-type and *bam1-2D* mutant plants (purple arrows). Free BAM1 is indicated with a green arrow. C, Native gel stained for MDH activity. Note that the position of the LSF1–MDH–BAM1 complex is different for wild-type and *bam1-2D* mutant plants (purple arrows) The LSF1–MDH complex is indicated with a black arrow, and free MDH with a green arrow. Gels were loaded so that each lane contained material from the same fresh weight of tissue.

We used this information to discover whether replacement of BAM1 with BAM1(S132N) in the *bam1-2D* mutant disrupts complexes formed between BAM1, LSF1, and pNAD-MDH (Figure  5; Supplemental Figure S2B). We confirmed that BAM1 activity was present in two bands on native amylopectin-containing gels of wild-type leaf extracts. The upper band represents BAM1 complexed with LSF1, and the lower band represents free BAM1 (Schreier et al., 2019; Figure  5A). As expected, both bands were absent from the *bam1-1* mutant and the upper band was absent from the *lsf1* mutant. In the *bam1-2D* mutant, the upper band was present but there was little or no BAM activity in the lower position on the gel. We concluded that BAM1(S132N) in *bam1-2D* mutants may be almost entirely complexed with LSF1. Surprisingly, although BAM1 protein was detected on immunoblots of SDS gels of *bam1-2D lsf1* extracts (Supplemental Figure S2A), repeated experiments failed to detect BAM1 activity on native gels of *bam1-2D lsf1* extracts.

These results were backed up by immunoblotting of native gels (without amylopectin) with BAM1 antiserum (Figure  5B). BAM1 protein was detected in the upper (BAM1-LSF1) and the lower (free BAM1) position on the gel from wild-type extracts, but only in the lower position from extracts of *lsf1* mutants. Protein was detected only in the lower position in extracts of *lsf1* mutants, and only in the upper position in extracts of *bam1-2D* mutants. The band from *bam1-2D* extracts migrated slightly faster than the upper band from wild-type extracts. Consistent with the failure to detect BAM1 activity on native gels of *bam1-2D lsf1* extracts, no BAM1 protein was detected in these extracts on blots of native gels.

Patterns of mobility of MDH activity on native gels (using the method described in Beeler et al., 2014) indicated that BAM1(S132N), like BAM1, can form complexes with both LSF1 and pNAD-MDH (Figure  5C). Three bands of MDH activity present in wild-type extracts were absent from extracts of a mutant with strongly reduced pNAD-MDH (*miR*-*mdh-*1; Beeler et al., 2014). The band with greatest electrophoretic mobility is likely to be free pNAD-MDH, since it was absent only from the *mdh* mutant. Both remaining bands were missing from the *lsf1* mutant, and one was missing from the *bam1-1* mutant. We concluded that the band with the lowest electrophoretic mobility represents a BAM1–LSF1–MDH complex, since it was missing from the *mdh*, *bam1-1*, and *lsf1* mutants. It was also missing from the *bam1-2D* mutant, but another band with slightly higher mobility was present. This may represent a BAM1(S132N)–LSF1–MDH complex. The remaining (middle) band of MDH activity was present in wild-type, *bam1-2D* and *bam1-1* but absent from the *mdh* and *lsf1* mutants. We conclude that this band represents an LSF1–MDH complex.

We also checked whether loss of LSF1 affected levels of BAM1 protein (Supplemental Figure S2). This was not the case: loss of LSF1 appeared to have little effect on levels of BAM1 in wild-type and *bam1-2D* mutant plants.

### Is BAM1(S132N) directly responsible for accelerated starch degradation?

**Figure 6:**
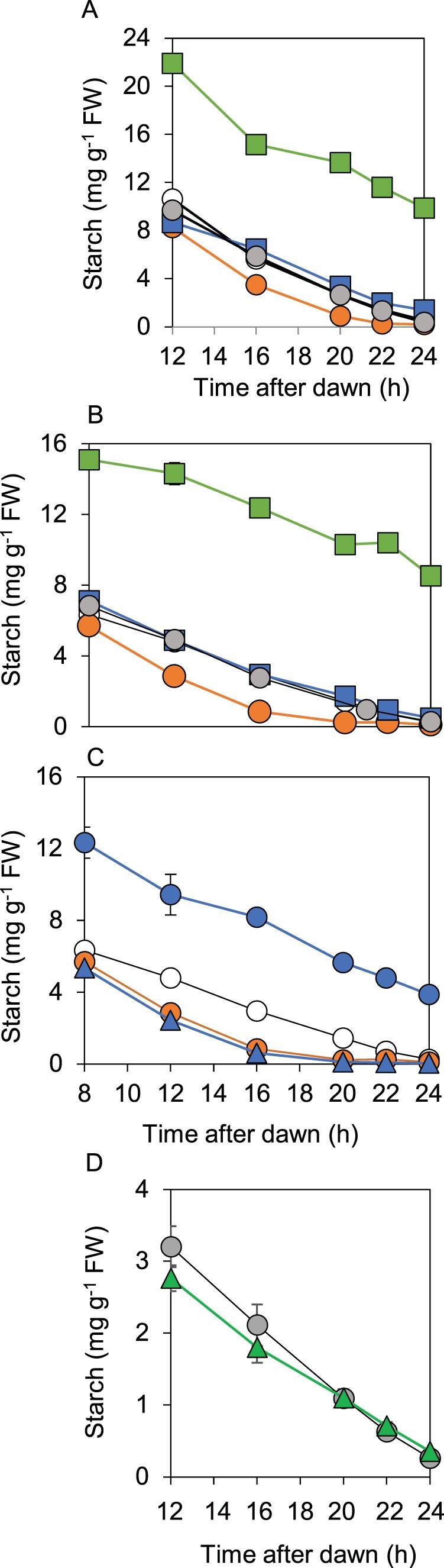
Comparison of patterns of starch degradation in wild-type plants, *bam1, lsf1*, and *bam3* mutants, and double mutants*.* Plants were grown with 12-h light, 12-h dark for 21 or 28 d. A, Starch **Figure 6:** (continued) degradation over a normal, 12-h night, comparing *bam1-2D*, *lsf1*, and *lsf1 bam1-2D* mutants. Open circle, wild-type. Gray circle, *bam1-1*. Orange circle, *bam1-2D*. Green square, *lsf1*. Blue square, *lsf1 bam1-2D*. B, As for (A) but showing starch degradation over an early night, starting at 8 h after dawn. C, As for (B) but comparing wild-type (open circle), *bam1-2D* (orange circle), *bam3* (blue circle), and *bam1-2D bam3* (blue triangle) mutants. D, Starch degradation over a normal, 12-h night, comparing *bam1-1* (gray circle) and *bam1-2D lsf1 bam3* (green triangle). Confirmatory data from a second, independently-selected *bam1-2D lsf1 bam3* triple mutant are shown in Supplemental Figure S5. Data are means of measurements on five individual rosettes. Error bars are se: where not visible, they are smaller than the symbol.

Although BAM1(S132N)–LSF1 complexes were formed in the *bam1-2D* mutant, analysis of patterns of starch degradation in *lsf1*, *bam1-2D*, and *bam1-2D lsf1* mutants revealed strong and unexpected interdependencies between these proteins (Figure  6). Progeny from a cross between the *lsf1* and *bam1-2D* mutants had a starch degradation phenotype different from that of either parent. The *bam1-2D lsf1* mutant did not display either the accelerated starch degradation of the *bam1-2D* mutant or the starch excess phenotype of the *lsf1* mutant. Instead, the rate of starch degradation was linear and was such that starch reserves were exhausted approximately at dawn. This was true in both a 12-h d, 12-h night and in response to an early night: in other words, the rate of starch degradation was correctly adjusted to the length of the night in the presence of BAM1(S132N) when LSF1 was absent (Figure  6, A and B). These data indicate that LSF1 is required for the accelerated rate of starch degradation brought about by BAM1(S132N), and also that normal starch degradation can be achieved without LSF1 when BAM1 is replaced by BAM1(S132N).

Our data thus far did not reveal whether BAM1(S132N) itself is responsible for the very fast starch degradation in the *bam1-2D* mutant, or whether the mutation results in loss of control of other enzymes essential for normal starch degradation. Accordingly, we examined whether BAM3 was necessary for the accelerated starch degradation in the *bam1-2D* mutant (Figure  6D; Supplemental Figure S5). BAM3 is essential for normal starch degradation, and is the major hydrolase involved in this process. Loss of BAM3, in the *bam3* mutant, results in a starch excess phenotype (Fulton et al., 2008). Like BAM1, BAM3 interacts with LSF1 (Schreier et al., 2019). We found that BAM3 was not necessary for the accelerated starch degradation seen in the *bam1-2D* mutant (Figure  6C). The double mutant *bam3 bam1-2D* had accelerated starch degradation, and was not distinguishable from the *bam1-2D* single mutant. BAM3 was also not required for the linear, wild-type-like starch degradation seen in the *bam1-2D lsf1* mutant: loss of BAM3 in this double mutant background (i.e. in the triple mutant *bam1-2D lsf1 bam3*) had no effect on the pattern of starch degradation.

We checked whether two further components of starch degradation were required for the accelerated rate seen when BAM1 was replaced by BAM1(S132N). BAM4 is catalytically inactive but is necessary for normal starch degradation; the *bam4* mutant, like the *bam3* mutant, has a strong starch excess phenotype (Fulton et al., 2008). The plastidial α-amylase AMY3 is not required for normal starch degradation, but can participate when other components of the pathway are missing (Yu et al., 2005; Streb et al., 2008, 2012; Seung et al., 2016).

We found that neither BAM4 nor AMY3 is required for the accelerated rate of starch degradation seen in the *bam1-2D* mutant (Supplemental Table S3). During the first eight hours of an early night, the double mutants *bam1-2D bam4* and *bam1-2D amy3* had accelerated rates of starch degradation, like *bam1-2D*.

### Function of S132 in the BAM1 protein

It was not obvious how the conversion of serine 132 to asparagine could bring about the observed dramatic changes in BAM1 properties. This substitution does not affect known substrate binding sites or catalytic site residues (described in Fulton et al., 2008). It is possible that the mutation alters posttranslational modification sites and thereby modulates enzyme activity. Phosphorylation of serine residues is a common posttranslational modification that can alter enzyme catalytic properties. Although Ser-132 was not found to be phosphorylated in previous phosphoproteomic analyses (Reiland et al., 2009; van Bentem et al., 2008), it remains possible that loss of this residue prevented an undetected phosphorylation/dephosphorylation essential for the regulation of enzyme activity.

**Figure 7:**
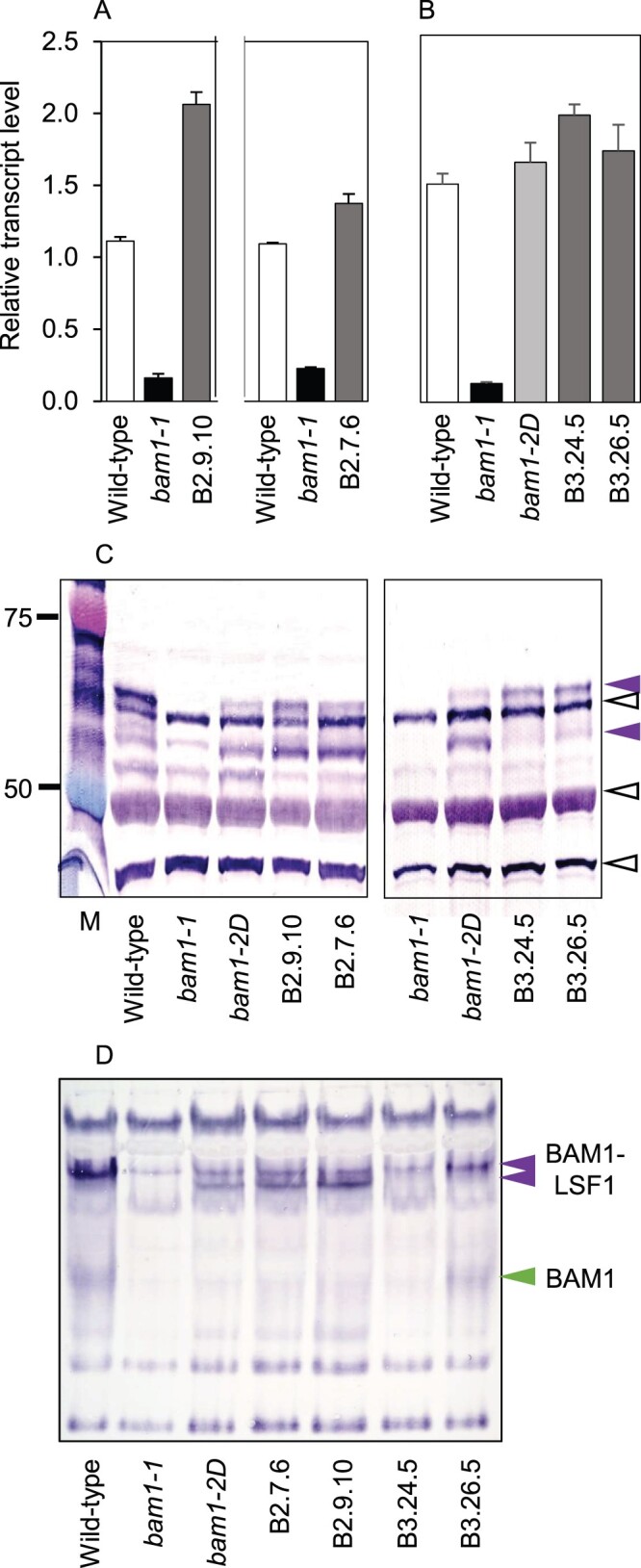
Transcript and protein abundance and native immunoblots for BAM1 in wild-type and mutant plants and transgenic plants expressing BAM1(S132**D**) or BAM1(S132**A**). A, Two independent experiments showing *BAM1* transcript levels in wild-type, *bam1-1* and two independent transgenic lines expressing BAM1(S132**D**) in a *bam1-1* background (lines B2.9.10 and B2.7.6). B, Transcript levels of wild-type, *bam1-1*, *bam1-2D* and two independent transgenic lines expressing BAM1(S132**A**) in a *bam1-1* background (lines B3.24.5 and **Figure 7:** (continued) B3.26.5). Levels are expressed relative to the level of *UBQ10* transcript. Values are means ± se of measurements made on three biological replicates. C, Immunoblot of a denaturing gel of extracts of the lines in (A), developed with BAM1 antiserum as for Figure  3B. Each lane was loaded with 2 μg protein from a soluble extract. Purple arrows indicate BAM1 protein, upper and lower bands (see Figure  3B); open black arrows indicate nonspecific bands. Note the absence of the lower band from transgenic lines expressing BAM1(S132**A**) (lines B3.24.5 and B3.26.5). D, Immunoblot of a native, amylose-containing gel of extracts of the lines in (A), developed with BAM1 antiserum. Each lane was loaded with 10 μg protein from a soluble extract. BAM1-LSF bands are marked with purple arrows. Note the presence of a lower BAM1-LSF1 band in *bam1-2D* and transgenic plants expressing BAM1(S132**D**) (B2 lines) but not BAM1(S132**A**) (B3 lines). The band of free BAM1 (green arrow) is visible in one but not the other BAM1(S132**A**) line on this blot. It was visible in both lines on other blots of the same extracts (Supplemental Figure S6).

As a first step toward discovering the significance of serine 132, we expressed constructs encoding BAM1 with either aspartate or alanine substituted for serine 132 (BAM1(S132**D**) and BAM1(S132**A**)) in the *bam1-1* mutant (Figure  7). These modifications could reveal whether serine 132 is a target for activity-modulating phosphorylation/dephosphorylation. Substitution of aspartate for serine residues that are subject to phosphorylation causes proteins to behave as if permanently phosphorylated, whereas substitution of alanine prevents phosphorylation (e.g. Gu et al., 2009). For both modified proteins, we selected two independent transgenic lines in which *BAM1* transcript levels were comparable with or a little higher than those of BAM1 transcript in wild-type plants.

Immunoblotting showed that, like *bam1-2D*, the transgenic lines expressing BAM1(S132**D**) had substantially lower levels of protein in the upper putative BAM1 band than wild-type plants, but more protein in the putative BAM1 lower band than wild-type plants (Figure  7C). In contrast, the transgenic lines expressing BAM1(S132**A**) gave bands in the position of the upper putative BAM1 band, but no band in the lower position. These data could be taken to imply that substitution of serine at position 132 by the polar amino acid aspartate had the same effect on the electrophoretic mobility of the denatured protein as substitution of the polar amino acid asparagine—as in the *bam1-2D* mutant—whereas substitution of the small, nonpolar amino acid alanine gave a protein with the same electrophoretic mobility as the wild-type protein.

This idea was strengthened by examination of immunoblots of native gels (Figure  7D; Supplemental Figure S6). Like *bam1-2D*, the transgenic lines expressing BAM1(S132**D**) had two low-mobility immunoreactive bands with similar electrophoretic mobility to BAM1 in complex with LSF1/pNAD-MDH. The transgenic lines expressing BAM1(S132**A**) had a single low-mobility band, in the same position as in wild-type. The band of high electrophoretic mobility representing free BAM1 was present in wild-type and in transgenic plants expressing BAM1(S132**A**), but not in *bam1-2D* or transgenic lines expressing BAM1(S132**D**).

**Figure 8:**
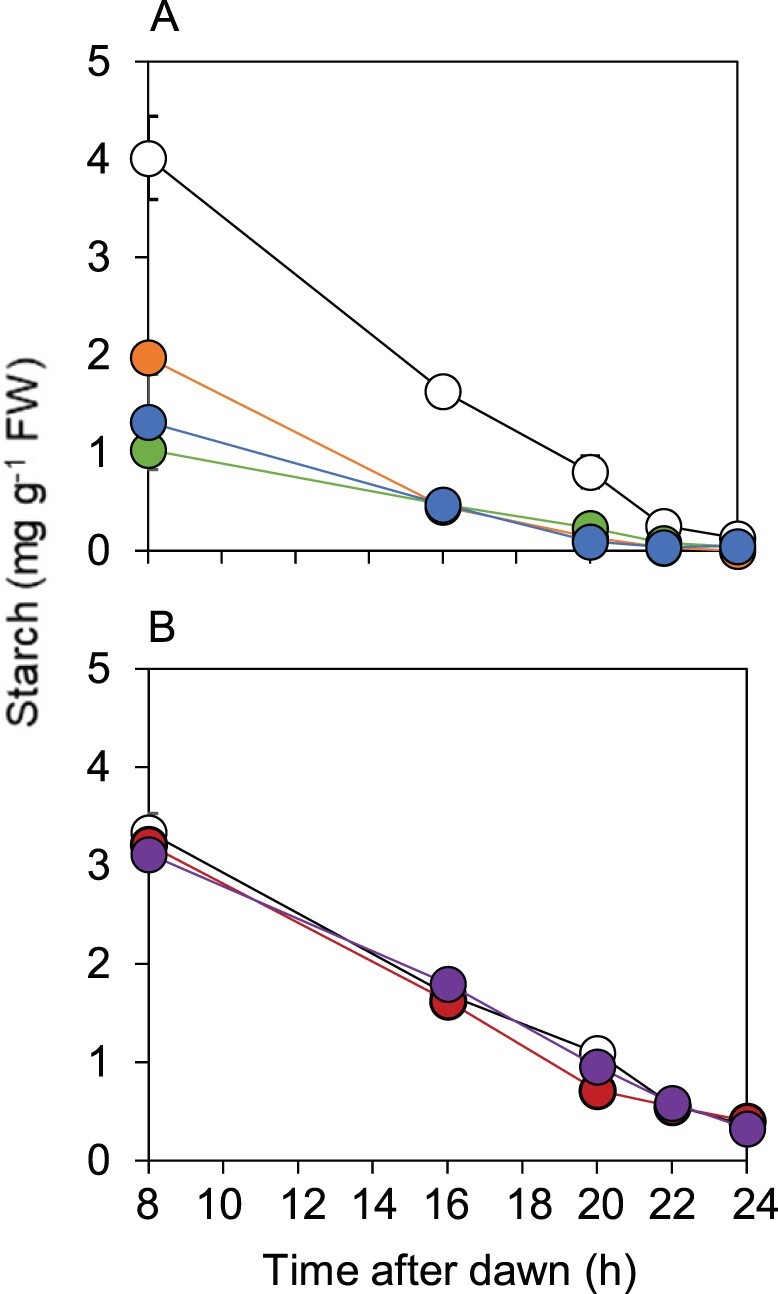
Effect of S132**D** and S132**A** substitutions in BAM1 on starch degradation. Plants were grown with 12-h light, 12-h dark for 21 d. Starch contents were measured over an early night, starting 8 h after dawn. A, Comparison of wild-type (open circle), *bam1-2D* (orange), and two lines expressing BAM1(S132**D**) in a *bam1-1* background: B2.7.6 (blue) and B2.9.10 (green). B, Comparison of wild-type (open circles) and two lines expressing BAM1(S132**A**) in a *bam1-1* background: B3.24.5 (red) and B3.26.5 (purple). Data are means of measurements on five individual rosettes. Error bars are se: where not visible, they are smaller than the symbol.

The rate of starch degradation in the transgenic lines expressing BAM1(S132**D**) was accelerated relative to that of wild-type plants, and similar to that of *bam1-2D* plants (Figure  8). The rate of degradation and absolute amounts of starch in the transgenic lines expressing BAM1(S132**A**) were not distinguishable from those of wild-type plants. Thus the mobility of native and denatured proteins and the pattern of starch degradation during the night resembled *bam1-2D* when S132 was substituted with aspartate, and resembled wild-type when S132 was substituted with alanine.

## Discussion

### BAM1(S132N) behaves very differently from BAM1 in vivo but not in vitro

We have established that a single amino acid change in the plastidial β-amylase BAM1 is sufficient to override the normal controls on night-time starch degradation in

Arabidopsis leaves and bring about an accelerated rate of degradation. In wild-type leaves, the rate of degradation is linear during the night and results in exhaustion of starch reserves at dawn (Graf et al., 2010). A complex mechanism maintains these properties in the face of changes in the timing of onset of the night, the amount of starch present, and the night-time temperature (see below; Pyl et al., 2012; Scialdone et al., 2013). The mechanism thus optimizes the fraction of daily photoassimilate allocated to growth under a wide range of external conditions (Sulpice et al., 2014). It appears that this mechanism can be disrupted by a change in the BAM1 protein.

Two features of the *bam1-2D* mutant—in which wild-type BAM1 is replaced with an S132N variant—are unexpected. First, the *bam1-2D* allele is dominant in the sense that a single *bam1-2D* allele is sufficient to bring about accelerated starch degradation in *bam1-2D BAM1* heterozygotes (based on the early expression of the starvation reporter in these plants). The introduction of BAM1(S132N) protein at near wild-type levels into the null mutant *bam1-1* gave an accelerated rate of degradation. Introduction of BAM1(S132N) into a wild-type background also accelerated starch degradation, but to a much lesser extent (Figure  4): a higher level of expression might have had a greater effect. Second, BAM1 is regarded as a minor isoform in Arabidopsis leaf chloroplasts. Its absence in a *bam1-1*, a null mutant, had no impact on starch degradation, total activity or growth. In a mutant possessing BAM1 but lacking all other plastidial BAMs (B1 quad), there was a mild starch excess phenotype (Monroe, 2020). In contrast, loss of the dominant isoform BAM3 in either a wild-type or a quad mutant background (B3 quad) markedly reduced total β-amylase activity and resulted in a strong starch-excess phenotype. Below we discuss how the S132N change in the BAM1 protein may bring about the observed strong and genetically dominant acceleration of starch degradation.

Neither the level of expression of BAM(S132N) nor the properties of the recombinant enzyme provide an explanation for its acceleration of starch degradation in vivo. Levels of *BAM1* transcript were about the same in wild-type and *bam1-2D* plants (Figure  3A). Recombinant BAM1 and BAM(S132N) proteins had near-identical properties with respect to specific activity, pH optimum, redox activation and thermal inactivation (Supplemental Figure S3). Levels of BAM1 protein detected by immunoblotting were considerably lower in the *bam1-2D* mutant than in wild-type plant, and the ratios of the upper and lower BAM1 bands were different (Figure  3B; Supplemental Figure S2). Protein sequencing suggested that neither the low abundance nor the altered mobility of the protein on SDS–polyacrylamide gel electrophoresis (PAGE) was due to proteolysis of BAM1(S132N). It seems likely therefore that the substitution of asparagine for serine at position 132 radically affects the behavior of the protein in vivo. Although it remains possible that S132 in the wild-type protein is subject to functionally important phosphorylation/dephosphorylation that is disrupted when asparagine is substituted, the results of our substitution experiments do not support this suggestion. Aspartate acts as a phosphomimetic because of its negative charge which mimics a phosphate group. Neither asparagine nor alanine has a negative charge, so are not phosphomimetics. The fact that both asparagine and aspartate substitutions cause a strong and similar phenotype whereas alanine does not, therefore suggests that altered phosphorylation potential at position 132 is not the cause of the strong phenotype. An alternative explanation may stem from structural considerations. Aspartate and asparagine are very similar in mass and considerably larger than serine and alanine, thus their substitution at position 132 may give rise to conformational changes within the BAM1 protein that affect its behavior in vivo.

Together, our observations indicate that BAM(S132N) behaves differently from BAM1 in vivo. As described below, our data suggest that the different behavior stems from altered interactions between BAM1 and other proteins necessary for the normal control of starch degradation. This idea is consistent with observations of Meinke (2013) on dominant mutations brought about by chemical mutagenesis: these tend to be associated not with elevated transcripts but with modified protein function with respect to protein–protein interactions.

### A BAM1(S132N)–LSF1 complex radically alters patterns of starch degradation

We pursued the possibility that the recently reported interaction between BAM1 and LSF1 (Schreier et al., 2019) might be physically and/or functionally disrupted in the *bam1-2D* mutant. We found abundant evidence that the complex was physically altered by substitution of BAM1(S132N) for wild-type BAM1. The band believed to represent activity of free BAM1 protein on amylopectin-containing native gels of wild-type leaves was absent from extracts of *bam1-2* leaves, raising the possibility that most or all of the BAM1(S132N) protein exists in a complex with LSF1. As in wild-type plants, this complex also contained the plastidial MDH pNAD-MDH. The mobility of the complex in native gels differed slightly between wild-type and *bam1-2D* extracts, consistent with alterations brought about by substitution of BAM1(S132N) for the wild-type BAM1 (Figure  5).

While loss of BAM1 protein had no effect on starch contents in either wild-type or *lsf1* mutant plants (Schreier et al., 2019), LSF1 was essential for the accelerated rate of starch degradation when BAM1(S132N) was substituted for wild-type BAM1. Loss of LSF1 in the presence of BAM1(S132N) (the *bam1-2D lsf1* mutant) prevented both the accelerated starch degradation of *bam1-2D* mutants and the starch excess phenotype of *lsf1* mutants and led to near-normal starch degradation—that is, rates that were both linear and appropriate for the length of the night (Figure  6). It is not clear how the presence of BAM1(S132N) overcomes the requirement for LSF1 for normal starch degradation, but it suggests that BAM1(S132N) influences the formation and function of complexes of starch-degrading enzymes in a manner that renders LSF1 redundant.

To investigate the importance of BAM1(S132N) further, we examined whether the accelerated rate of starch degradation in *bam1-2D* mutants required the presence of other components of the starch degradation machinery, in particular BAM3, the major BAM activity in the chloroplast. It seemed reasonable to propose that BAM3 rather than BAM1(S132N) might be responsible for or at least contribute to the accelerated starch degradation in *bam1-2D* given the low levels of BAM1(S132N) protein and the known association of BAM3 as well as BAM1 with the LSF1 complex (Schreier et al., 2019). However, BAM3 was not required for either the accelerated starch degradation in *bam1-2D* mutants or the near-normal pattern of starch degradation in *bam1-2D lsf1* mutants (Figure  6C). Further genetic analysis showed that the accelerated starch degradation required neither BAM4 nor the α-amylase AMY3. Thus the accelerated rate is not due to altered regulation of the activity of the major BAM of the chloroplast, or a noncatalytic BAM that is necessary for starch degradation, or the only plastidial α-amylase. The remaining amylases of the chloroplast, BAM2 and BAM6, appear to have very low activity and little or no influence on starch turnover (Fulton et al., 2008; Monroe, 2020), but the possibility of involvement of one or both in starch degradation in *bam1-2D* cannot be ruled out. Taken as a whole, however, these results suggest that BAM1(S132N) itself is responsible for accelerated rates when complexed with LSF1 in vivo (Figure  6).

### BAM1(S132N) influences the phenotypes of other starch-degradation mutants

Given that BAM3 is responsible for most of the plastidial β-amylase activity in the leaf and its loss leads to major starch accumulation in a wild-type background, it is perhaps surprising that it is not necessary for the accelerated starch degradation in the presence of BAM1(S132N). However, although loss of BAM3 leads to a starch-excess phenotype, it has relatively little effect on the rate of degradation. The same is true for starch-excess mutants lacking several other components of the starch degradation pathway. For example, rates of leaf starch degradation estimated from published data for *bam3* are ∼ 82%, 125% and 125% of wild-type rates (data displays in Fulton et al., 2008; Schreier et al. 2019; see also Figure  6C). The same applies to *bam3 bam1* mutants (73% and 106% of wild-type rates) and *bam3 bam4* mutants (105% and 96% of wild-type rates) (data from Fulton et al., 2008; Schreier et al. 2019). These data imply that starch-excess phenotypes do not stem directly from a loss of starch-degrading capacity, but rather from a failure of the system-level mechanism proposed to control the rate of degradation. In the arithmetic division model of the control of starch degradation, the rate needed to consume by dawn all of the starch present at the end of the day is computed by division of starch content by the time remaining to dawn. Time to dawn is represented by a hypothetical entity T, generated by the circadian clock, and starch content is represented by a hypothetical entity S, concentration of which precisely tracks starch content so that S and starch are both consumed by dawn. The model gives rise to the prediction that if the relationship between the consumption of S and the actual consumption of starch is perturbed—in other word changes in S fail to track changes in starch—the resulting rate of degradation will not allow consumption of all of the starch precisely at dawn (Scialdone et al., 2013). This phenomenon is seen in *lsf1* and *bam3* mutants, for example, which have rates of degradation comparable with wild-type but consume only ∼50% of end-of-day starch by dawn (Schreier et al., 2019).

In the light of the arithmetic division model, we can speculate that the substitution of BAM1(S132N) for BAM1 strongly disrupts the relationship between the hypothetical entity S and the actual starch content when LSF1 is present. Starch content in the *bam1-2D* mutant is perceived as larger than it actually is, resulting in accelerated degradation. In the absence of LSF1, BAM1(S132N) permits correct computation of the timing of degradation even though loss of LSF1 would lead to incorrect computation in a wild-type background. At present, we have insufficient information to resolve the mode of action of BAM1(S132N). However, the striking changes in the pattern of starch degradation brought about by this single amino acid change strengthen the view that control of degradation in wild-type plants is brought about by a sophisticated mechanism that requires dynamic interactions of several degradative enzymes and related proteins. LSF1 complexes in particular appear to be essential for the normal control of starch degradation.

The loss of control of the rate of starch degradation seen in the presence of BAM1(S132N) is superficially similar to the phenomenon we observed previously in mutants lacking the granule-bound protein ESV1 (Feike et al., 2016). In both cases, the rate of degradation is accelerated so that starch reserves are depleted before dawn, and the rate of degradation is not adjusted to changes in day length. However, the mechanisms underlying these effects differ profoundly. The S132N mutation in BAM1 is dominant, and its acceleration of starch degradation does not require other hydrolytic and related enzymes. The acceleration does however require the complex-forming protein LSF1. In contrast, mutations in ESV1 are recessive. The acceleration of degradation in the absence of ESV1 is partially dependent on BAM3 and BAM4 as well as on LSF1. Based on these observations, we propose that whereas loss of ESV1 affects the degradation control mechanism at the granule surface, replacement of BAM1 with BAM1(S132N) affects downstream parts of the control mechanism. Further study of BAM1 and BAM1 variants alongside ESV1 holds promise for the full elucidation of the control mechanism.

## Materials and methods

### Plant material

Arabidopsis (*A.* *thaliana)* plants were in a Col-0 background except where Ler was used in mapping experiments. The *bam1-2D* mutant was isolated from a mutagenized starvation reporter line expressing pAt1g10070:LUC, using methods described by Feike et al. (2016). The mutant was crossed to *Ler* and F2 plants were analyzed for luminescence at the end of an early night. Since the mutation was dominant, homozygous and heterozygous mutants could not be distinguished. Therefore, DNA was extracted from F2 plants that did not exhibit luminescence at the end of an early night, that is, carried only the wild-type allele of the target gene originating from the *Ler* parent. Mapping pinpointed a region of 1.4 Mb containing 453 genes on chromosome 3. The only gene in the interval known to be related to starch metabolism was *BAM1* (At3g23920). Sequencing revealed an EMS-induced point mutation in the Col-0 allele. Other mutants used in this study were T-DNA insertion lines described previously, as follows: *bam1-1* (Salk_039895), *bam3* (At4g17090; CS92461), and *bam4* (At5g55700; SALK_037355) (Fulton et al., 2008); *lsf1-1* (At3g01510; SALK_053285; Comparot-Moss et al., 2010); *amy3-2* (At1g69830; SAIL_613 D12; Yu et al., 2005); *sex4-3* (At3g52180; SALK_102567; Niittylä et al., 2006); and a line depleted in pdNAD-MDH (At3g47520; *miR*-*mdh-*1) described by Beeler et al. (2014).

Except where otherwise stated, plants were grown on soil with 12-h light, 12-h dark, 20°C and 75% relative humidity either in controlled environment rooms (light intensity ∼100 μmol quanta m^−2^ s^−1^) or growth cabinets (light intensity 150–180 μmol quanta m^−2^ s^−1^. Plants grown at 100 μmol quanta m^−2^ s^−1^ had lower end-of-day starch contents than those grown at higher light levels, but the patterns of starch degradation described in the text were the same at the two light levels.

### Generation of double and triple mutants

Double mutants were generated by cross-pollinating the respective homozygous single mutants and allowing self-pollination of the F1 generation. For detection of suitable lines, polymerase chain reaction (PCR) amplification was carried out using primers in Supplemental Table S4. The mutation in *bam3-1* was determined by PCR amplification followed by restriction enzyme digestion as in Fulton et al. (2008). For triple mutants, *bam1-2 lsf1* was crossed with *bam1-2 bam3-1* and F2 progeny were selected for the *lsf1* and *bam3-1* mutations.

### Plant transformation

*BAM1* and *bam1-2D* coding sequences including start and stop codons were generated from Col-0 or *bam1-2D* cDNA by PCR using Platinum high-fidelity DNA polymerase (Invitrogen: thermofisher.com). Site-directed mutagenesis (Q5 Mutagenesis, New England BioLabs: neb.com) of *BAM1* cDNA was used to convert Ser132 into Ala or Asp. Primers used are given in Supplemental Table S4.

Fragments were cloned using the cloning kit pCR8/GW/TOPO/TA (Invitrogen). Plasmids were sequenced using primers listed in Supplemental Table S4. Using Clonase II in the LR reaction, inserts were transferred into the Destination vector pGWB2, a Gateway Binary vector for the expression of proteins in plants driven by a 35S promoter (Nakagawa et al., 2007).

pGWB2 constructs were transformed into electrocompetent *Agrobacterium tumefaciens* strain Agl1. Arabidopsis plants were transformed using the floral dip method (Zhang et al., 2006). Seeds were sterilized with chlorine gas then screened on 0.8% (w/v) agar plates containing MS + 1% (w/v) sucrose, 20-μg mL^**−**1^ nystatin, 50-μg mL^**−**1^ kanamycin and 50-μg mL^**−**1^ hygromycin. After 48 h in the dark at 4**°**C, plates were incubated with 16**-**h light (150 μmol quanta m^**−**2^ s^**−**1^) 8-h dark at 20°C. Transformed seedlings were selected at 10–14 d and transferred to soil. PCR was used to check for correct insertions in T1 and T2 plants.

### RT-qPCR analysis

mRNA was extracted from single, 19-d-old rosettes harvested after 4 h light using the RNeasy kit (Qiagen: quiagen.com). Following Dnase treatment, 2-μg total RNA was used for cDNA synthesis with the SuperScript IV kit (Invitrogen). RT-qPCR was performed using the SYBRgreen PCR kit (Qiagen) and a DNA Engine Opticon 4 system (MJ Research: mj-research.com). Actin primer sets were used to check for gDNA contamination. Primer pair sequences are given in Supplemental Table S4. Threshold cycle values were determined using MJ Opticon Monitor software version 3.1 (Bio-Rad: bio-rad.com) normalized against the *UBIQUITIN 10* or YSL8 housekeeping genes.

### Gel electrophoresis and immunoblotting

Extracts were prepared from frozen leaf tissue homogenized in 100-mM MOPS (pH 7.2), 1-mM ethylenediaminetetraacetic acid (EDTA), 2 mM DTT, 10% (v/v) ethanediol at a tissue:medium ratio of 1:6–1:4 (w/v). After centrifugation at 16,000*g*, soluble fractions were mixed with the appropriate gel loading buffers. Gels were loaded on an equal tissue fresh weight or a protein basis.

Native gels were 7.5% or 10% polyacrylamide unless otherwise stated. Activity gels contained 1% (w/v) amylopectin except for those for MDH activity which were performed according to Beeler et al. (2014). Native gels for activity were incubated in 100 mM Tris–HCl pH 7.0, 1 mM MgCl_2_ 1 mM CaCl_2_ for ∼2 h at 37°C before staining with iodine. Native gels for immunoblotting contained either no glucan or ∼1 mg mL^−1^ amylose, added from an 8 mg mL^−1^ solution created by dispersion in ethanol followed by boiling in 2% (w/v) NaOH. For immunoblotting, SDS–polyacrylamide gels (4–12% acrylamide-gradient Nu-PAGE [Invitrogen], run in strongly reducing conditions throughout), and native gels following incubation in an SDS solution, were blotted onto PVDF membrane and probed with a specific antiserum for BAM1 (affinity purified as described in Thalmann et al. (2016) at a dilution of 1/1,200 except where otherwise stated).

### Peptide sequencing

Protein for peptide analysis was generated by immunoprecipitation from extracts of leaves of 28-d-old plants, homogenized in 50 mM Tris–HCl, pH 8, 150 mM NaCl, 1% (v/v) Triton X-100 and EDTA-free Protease Inhibitor (Sigma: sigmaaldrich.com) then clarified by centrifugation. Extracts were incubated with Protein A Sepharose beads and purified BAM1 antiserum. The precipitate was subjected to SDS–PAGE. Protein from excised bands was prepared for analysis as in Shevchenko et al. (2006), and analysis was by liquid chromatography mass Spec/Mass Spec (LC-MS/MS) essentially as in Andriotis et al. (2016).

### Starch content and composition

Starch content was assayed as glucose released by α-amylase and amyloglucosidase from the insoluble fraction of perchloric acid extracts of tissue following rapid freezing and homogenization in liquid nitrogen, as described previously (Smith and Zeeman, 2006).

### Expression of BAM1 in *E. coli* and characterization of recombinant proteins

BAM1 and BAM1(S132N) were recombinantly expressed and purified using *E.* *coli* BL21 (DE3) cells as in Sparla et al. (2006). At optical density 0.6 (600 nm) recombinant protein expression was induced with 0.4-mM isopropyl β-d-1-thiogalactopyranoside and cells were collected by centrifugation 4 h later. After sonication and centrifugation, supernatant was applied to a HisTrap HP column (GE Healthcare: gehealthcare.com). The resin was washed with 0.5 mM NaCl, 20 mM Tris–HCl pH 7.9 containing 5-mM imidazole, then 60 mM imidazole, then eluted with 250 mM imidazole in the same medium, desalted into 50-mM K phosphate buffer (pH 7.5), and incubated with thrombin (GE Healthcare) to remove the his-tag following the manufacturer’s instructions. Experiments were performed on two, independently prepared batches of recombinant protein, with essentially identical results. Data from one of the batches are displayed in Supplemental Figure S3.

Activity was assayed using the Betamyl 3 kit (Megazyme: megazyme.com). Thermostability was assessed by heating recombinant protein (0.125 mg mL^−1^, in 25 mM phosphate buffer pH 7.5) in a Peltier heating block for 20 min at the specified temperatures prior to assay. pH dependence assessments used 100-mM MES and Tris buffers for pH 5.5–6.5 and 7.0–9.0, respectively. Redox sensitivity was tested by incubating recombinant protein with 40-mM dithiothreitol (reduced DTT) or 80 mM trans-4,5-dihydroxy-1,2-dithiane (oxidized DTT) for 1 h at 37°C prior to assay.

### Accession numbers

Sequence data from this article can be found in the GenBank data library under accession number Gene ID 821975 (BAM1).

## Supplemental data

The following materials are available in the online version of this article.

**Supplemental Figure S1**. Relative rates of starch degradation in the wild-type and *bam1* mutants.

**Supplemental Figure S2**. BAM1 protein and activity in leaves of the wild-type and *bam1-2D* and *lsf1* single and double mutants.

**Supplemental Figure S3**. Properties of recombinant BAM1.

**Supplemental Figure S4**. Relative rates of starch degradation in *bam1-1* and *bam1-2D* plants and transgenic lines expressing BAM1(S132N).

**Supplemental Figure S5**. Comparison of patterns of starch degradation in *bam1-2D* and a *bam1-2D lsf1 bam3* mutant.

**Supplemental Figure S6**. Immunoblot of a native gel of transformants expressing modified BAM1 proteins.

**Supplemental Table S1.** Peptides recovered from BAM1 proteins.

**Supplemental Table S2.** Relative rates of starch degradation in the first 8 h of an early night in *bam1-1* and *bam1-2D* plants and transgenic lines expressing BAM1(S132N).

**Supplemental Table S3**. Starch consumption in the first 8 h of an early night in the wild-type and single and double mutant lines.

**Supplemental Table S4**. Primers used in this study.

## Supplementary Material

kiab603_Supplementary_Data

## Notes

A.G., D.F., M.P., and A.M.S. conceived the research plans and designed the experiments. D.F. and M.P. conducted the experiments and analyzed the data, with specific contributions from L.G. A.M.S. wrote the manuscript, with input from all the authors. All the authors proofread and approved the manuscript.

The author responsible for distribution of materials integral to the findings presented in this article in accordance with the policy described in the Instructions for Authors (https://academic.oup.com/plphys/pages/general-instructions) is: Alison M. Smith (alison.smith@jic.ac.uk).

## References

[kiab603-B1] Andriotis VME , Saalbach G, Waugh R, Field RA, Smith AM (2016) The maltase involved in starch metabolism in barley endosperm is encoded by a single gene. PLoS One 11: e0151642 27011041 10.1371/journal.pone.0151642 PMC4807107

[kiab603-B2] Beeler S , Lu HC, Stadler M, Schreier T, Eicke S, Lue WL, Truernit E, Zeeman SC, Chen J, Kötting O (2014) Plastidial NAD-dependent malate dehydrogenase is critical for embryo development and heterotrophic metabolism in Arabidopsis. Plant Physiol 164: 1175–1190 24453164 10.1104/pp.113.233866 PMC3938612

[kiab603-B3] Comparot-Moss S , Kötting O, Stettler M, Edner C, Graf A, Weise S, Streb S, Lue WL, MacLean D, Mahlow S, et al (2010) A putative phosphatase, LSF1, is required for normal starch turnover in Arabidopsis leaves. Plant Physiol 152: 685–697 20018601 10.1104/pp.109.148981 PMC2815883

[kiab603-B4] Feike D , Seung D, Graf A, Bischof S, Ellick T, Coiro M, Soyk S, Eike S, Mettler-Altmann T, Lu KJ, et al (2016) The starch granule-associated protein EARLY STARVATION1 is required for the control of starch degradation in *Arabidopsis thaliana* leaves. Plant Cell 28: 1472–1489 27207856 10.1105/tpc.16.00011 PMC4944407

[kiab603-B5] Fulton DC , Stettler M, Mettler T, Vaughan CK, Li J, Francisco P, Gil M, Reinhold H, Eicke S, Messerli G, et al (2008) β-AMYLASE4, a noncatalytic protein required for starch breakdown, acts upstream of three active β-amylases in Arabidopsis chloroplasts. Plant Cell 20: 1040–1058 18390594 10.1105/tpc.107.056507 PMC2390740

[kiab603-B6] Graf A , Schlereth A, Stitt M, Smith AM (2010) Circadian control of carbohydrate availability for growth in Arabidopsis plants at night. Proc Natl Acad Sci USA 107: 9458–9463 20439704 10.1073/pnas.0914299107 PMC2889127

[kiab603-B7] Gu X , Greiner ER, Mishra R, Kodali R, Osmand A, Finkbeiner S, Steffan JS, Thompson LM, Wetzel R, Yang W (2009) Serines 13 and 16 are critical determinants of full-length human mutant huntingtin induced disease pathogenesis in HD mice. Neuron 64: 828–840 20064390 10.1016/j.neuron.2009.11.020 PMC2807408

[kiab603-B8] Horrer D , Flütsch S, Pazmino D, Matthews JSA, Thalmann M, Nigro A, Leonhardt N, Lawson T, Santelia D (2016) Blue light induces a distinct starch degradation pathway in guard cells for stomatal opening. Curr Biol 26: 362–370 26774787 10.1016/j.cub.2015.12.036

[kiab603-B9] Kötting O , Santelia D, Edner C, Eicke S, Marthaler T, Gentry MS, Comparot-Moss S, Chen J, Smith AM, Steup M, et al (2009) STARCH-EXCESS4 is a laforin-like phosphoglucan phosphatase required for starch degradation in *Arabidopsis thaliana*. Plant Cell 21: 334–346 19141707 10.1105/tpc.108.064360 PMC2648081

[kiab603-B10] Meinke DW (2013) A survey of dominant mutations in *Arabidopsis thaliana*. Trends Plant Sci 18: 1360–1385 10.1016/j.tplants.2012.08.006 22995285

[kiab603-B11] Monroe JD (2020) Involvement of five catalytically active Arabidopsis β‐amylases in leaf starch metabolism and plant growth. Plant Direct 4: 1–10 10.1002/pld3.199 PMC7011640 32072133

[kiab603-B12] Nakagawa T , Kurose T, Hino T, Tanaka K, Kawamukai M, Niwa Y, Toyooka K, Matsuoka K, Jinbo T, Kimura T (2007) Development of series of gateway binary vectors, pGWBs, for realizing efficient construction of fusion genes for plant transformation. J Biosci Bioeng 104: 34–41 17697981 10.1263/jbb.104.34

[kiab603-B13] Niittylä T , Comparot-Moss S, Lue WL, Messerli G, Trevisan M, Seymour MDJ, Gatehouse JA, Villadsen D, Smith SM, Chen J, et al (2006) Similar protein phosphatases control starch metabolism in plants and glycogen metabolism in mammals. J Biol Chem 281: 11815–11818 16513634 10.1074/jbc.M600519200

[kiab603-B14] Pyl ET , Piques M, Ivakov A, Schulze W, Ishihara H, Stitt M, Sulpice R (2012) Metabolism and growth in Arabidopsis depend on the daytime temperature but are temperature-compensated against cool nights. Plant Cell 24: 2443–2469 22739829 10.1105/tpc.112.097188 PMC3406903

[kiab603-B15] Reiland S , Messerli G, Baerenfaller K, Gerrits B, Endler A, Grossmann J, Gruissem W, Baginsky S (2009) Large-scale Arabidopsis phosphoproteome profiling reveals novel chloroplast kinase substrates and phosphorylation networks. Plant Physiol 150: 889–903 19376835 10.1104/pp.109.138677 PMC2689975

[kiab603-B16] Santelia D , Kötting O, Seung D, Schubert M, Thalmann M, Bischof S, Meekins DA, Lutz A, Patron N, Gentry MS, et al (2011) The phosphoglucan phosphatase Like Sex Four2 dephosphorylates starch at the C3-position in Arabidopsis. Plant Cell 23: 4096–4111 22100529 10.1105/tpc.111.092155 PMC3246334

[kiab603-B17] Schreier TB , Cléry A, Schläfli M, Galbier F, Stadler M, Demarsy E, Albertini D, Maier BA, Kessler F, Hörtensteiner S, et al (2018) Plastidial NAD-dependent malate dehydrogenase: a moonlighting protein involved in early chloroplast development through its interaction with an FtsH12-FtsHi protease complex. Plant Cell 30: 1745–1769 29934433 10.1105/tpc.18.00121 PMC6139691

[kiab603-B18] Schreier TB , Umhang M, Lee SK, Lue WL, Shen Z, Silver D, Graf A, Müller A, Eicke S, Stadler-Waibel M, et al (2019) LIKE SEX4 1 acts as a β-amylase-binding scaffold on starch granules during starch degradation. Plant Cell 31: 2169–2186 31266901 10.1105/tpc.19.00089 PMC6751131

[kiab603-B19] Scialdone A , Mugford ST, Feike D, Skeffington A, Borrill P, Graf A, Smith AM, Howard M (2013) Arabidopsis plants perform arithmetic division to prevent starvation at night. eLife 2: e00669 23805380 10.7554/eLife.00669 PMC3691572

[kiab603-B20] Seung D , Lu KJ, Stettler M, Streb S, Zeeman SC (2016) Degradation of glucan primers in the absence of starch synthase 4 disrupts starch granule initiation in Arabidopsis. J Biol Chem 291: 20718–20728 27458017 10.1074/jbc.M116.730648 PMC5034061

[kiab603-B22] Shevchenko A , Tomas H, Havli J, Olsen JV, Mann M (2006) In-gel digestion for mass spectrometric characterization of proteins and proteomes. Nat Protoc 1: 2856–2860 17406544 10.1038/nprot.2006.468

[kiab603-B23] Smith AM , Stitt M (2007) Coordination of carbon supply and plant growth. Plant Cell Environ 30: 1126–1149 17661751 10.1111/j.1365-3040.2007.01708.x

[kiab603-B24] Smith AM , Zeeman SC (2006) Quantification of starch in plant tissues. Nat Protoc 1: 1342–1345 10.1038/nprot.2006.232 17406420

[kiab603-B25] Smith AM , Zeeman SC (2020) Starch: a flexible, adaptable carbon store coupled to plant growth. Annu Rev Plant Biol 71: 217–245 32075407 10.1146/annurev-arplant-050718-100241

[kiab603-B26] Sparla F , Costa A, Lo Schiavo F, Pupillo P, Trost P (2006) Redox regulation of a novel plastid-targeted β-amylase of Arabidopsis. Plant Physiol 141: 840–850 16698902 10.1104/pp.106.079186 PMC1489908

[kiab603-B27] Streb S , Delatte T, Umhang M, Eicke S, Schorderet M, Reinhardt D, Zeeman SC (2008) Starch granule biosynthesis in Arabidopsis is abolished by removal of all debranching enzymes but restored by the subsequent removal of an endoamylase. Plant Cell 20: 3448–3466 19074683 10.1105/tpc.108.063487 PMC2630441

[kiab603-B28] Streb S , Eicke S, Zeeman SC (2012) The simultaneous abolition of three starch hydrolases blocks transient starch breakdown in Arabidopsis. J Biol Chem 287: 41745–41756 23019330 10.1074/jbc.M112.395244 PMC3516724

[kiab603-B29] Sulpice R , Flis A, Ivakov AA, Apelt F, Krohn N, Encke B, Abel C, Feil R, Lunn JE, Stitt M (2014) Arabidopsis coordinates the diurnal regulation of carbon allocation and growth across a wide range of photoperiods. Mol Plant 7: 137–155 24121291 10.1093/mp/sst127

[kiab603-B30] Thalmann T , Pazmino D, Seung D, Horrer D, Nigro A, Meier T, Kölling K, Pfeifhofer HW, Zeeman SC, Santelia D (2016) Regulation of leaf starch degradation by abscisic acid is important for osmotic stress tolerance in plants. Plant Cell 28: 1860–1878 27436713 10.1105/tpc.16.00143 PMC5006701

[kiab603-B31] van Bentem SF , Anrather D, Dohnal A, Roitinger E, Csaszar E, Joore J, Buijnink J, Carreri A, Forzani C, Lorkovic ZJ, et al (2008) Site-specific phosphorylation profiling of Arabidopsis proteins by mass spectrometry and peptide chip analysis. J Proteome Res 7: 2458–2470 18433157 10.1021/pr8000173

[kiab603-B32] Yu TS , Zeeman SC, Thorneycroft D, Fulton DC, Dunstan H, Lue WL, Hegemann B, Tung SY, Umemoto T, Chapple A, et al (2005) α-Amylase is not required for breakdown of transitory starch in Arabidopsis leaves. J Biol Chem 280: 9773–9779 15637061 10.1074/jbc.M413638200

[kiab603-B33] Zanella M , Borghi GL, Pirone C, Thalmann M, Pazmino D, Costa A, Santelia D, Trost P, Sparla F (2016) β-amylase 1 (BAM1) degrades transitory starch to sustain proline biosynthesis during drought stress. J Exp Bot 67: 1819–1826 26792489 10.1093/jxb/erv572

[kiab603-B34] Zhang X , Henriques R, Lin SS, Niu QW, Chua NH (2006) Agrobacterium-mediated transformation of *Arabidopsis thaliana* using the floral dip method. Nat Protoc 1: 641–646 17406292 10.1038/nprot.2006.97

