# Supplementary material for: A dominant mutation in β-AMYLASE1 disrupts nighttime control of starch degradation in Arabidopsis leaves
Source: Plant Physiol. 2021 Dec 27;188(4):1979–92. doi: 10.1093/plphys/kiab603 (PMC8968401; doi:10.1093/plphys/kiab603)
Supplement: kiab603_Supplementary_Data [file kiab603_supplementary_data.pdf]

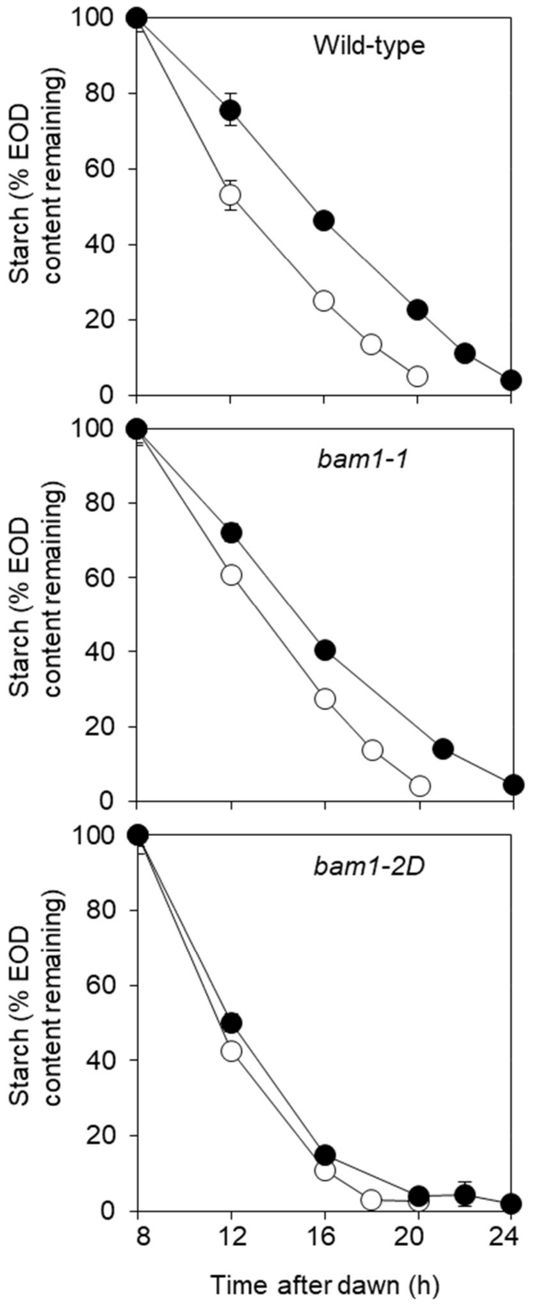

Supplemental Figure S1. Relative rates of starch degradation in the wild-type and *bam1* mutants. Data from Figure 2 are replotted as the % of end-of-day (EOD) starch remaining at intervals during the night, for a normal night of 12 h (open symbols) and an early night starting at 8 h after dawn (closed symbols). Data are means of measurements on five individual rosettes. Error bars are SE: where not visible, they are smaller than the symbol. Both wild-type and *bam1-1* adjust starch degradation in response to an early night; *bam1-2D* does not.

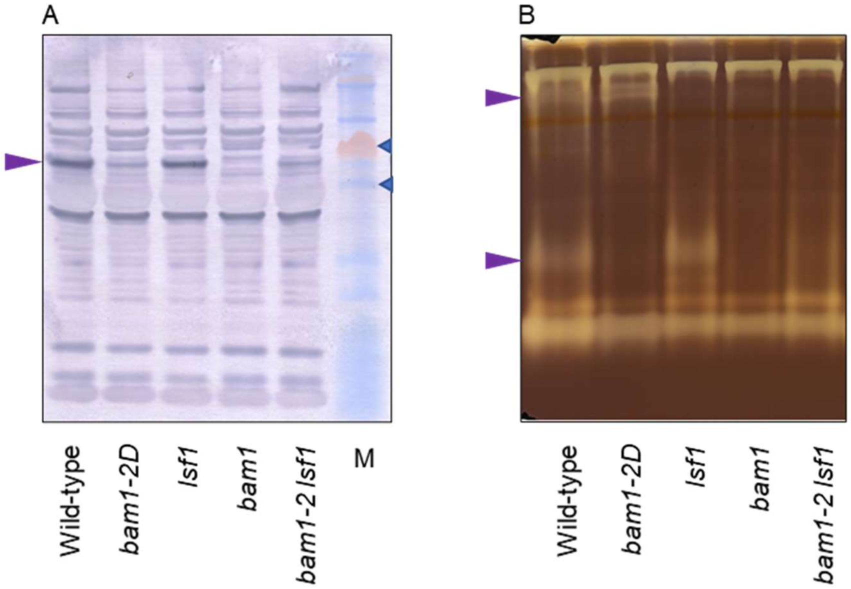

Supplemental Figure S2. BAM1 protein and activity in leaves of the wild-type and *bam1-2D* and *lsf1* single and double mutants. A, Immunoblot of an SDS 4-12% polyacrylamide gradient gel showing extracts from wild-type plants and *bam1-2D*, *lsf1* and *bam1-2D lsf1* mutants. The blot was probed with the BAM1 antiserum described by Fulton et al (2008). Under these conditions, there is little or no separation of the upper and lower bands of BAM1. A purple arrow indicates the position of BAM1. M is molecular mass markers: small arrows indicate 71 kD (upper arrow) and 48 kD (lower arrow) markers. All lanes contain extract derived from the same fresh weight of tissue. Extracts are from a different batch of plants from those used in Figure 5A. Note that the BAM1 band is much weaker in *bam1-2D* and in *bam1-2D lsf1* extracts than in wild-type extracts, and absent from *bam1-1* extracts.

B, Native gel containing amylopectin, stained with iodine to reveal starch-degrading activities. The identity of arrowed bands is deduced from Schreier et al. (2019) and from the presence/absence of the bands in different mutants. Extracts are from a different batch of plants from those used in Figure 5A.

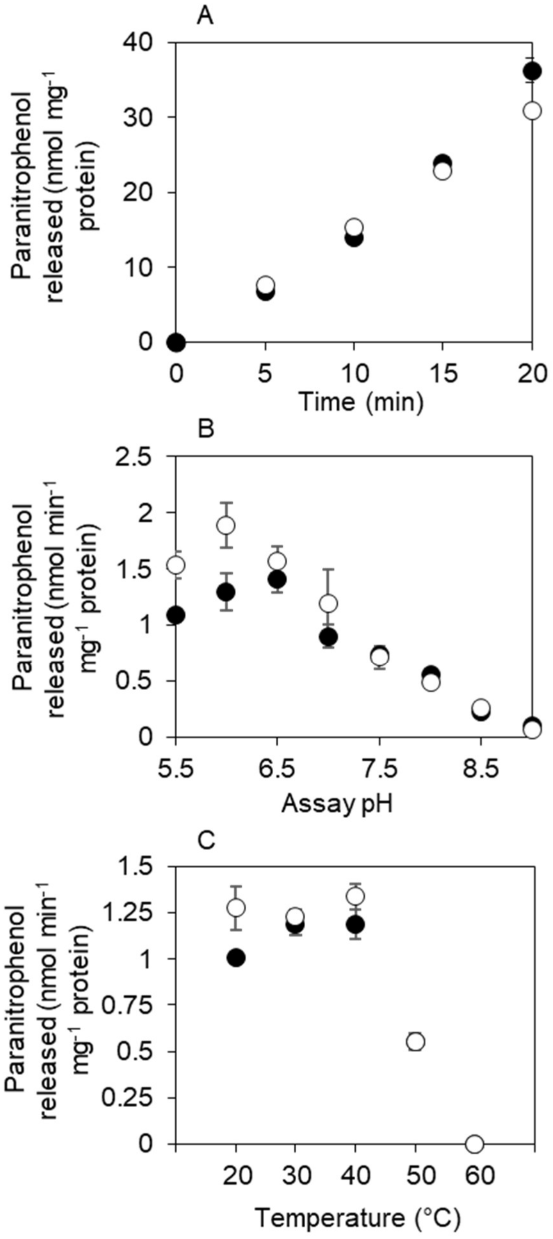

Supplemental Figure S3. Properties of recombinant BAM1. BAM1 and BAM1(S132N) were expressed as his-tag fusions in *E. coli*. After purification on a nickel column, his tags were removed by treatment with 1U thrombin per mg of recombinant protein prior to assay. Activity was measured at 37°C as the rate of release of paranitrophenol from p-nitrophenyl-β-D-maltotrioside in the presence of β-glucosidase. Black symbols, BAM1. Open symbols, BAM1(S132N). Values are means ± SD of measurements on three technical replicates. A, Linearity of paranitrophenol release over 20 min. B, Effect of pH from 5.5 to 9.0 on enzyme activity. C, Thermostability of activity. Purified enzyme was incubated for 20 min at the stated temperatures prior to assay at 37°C.

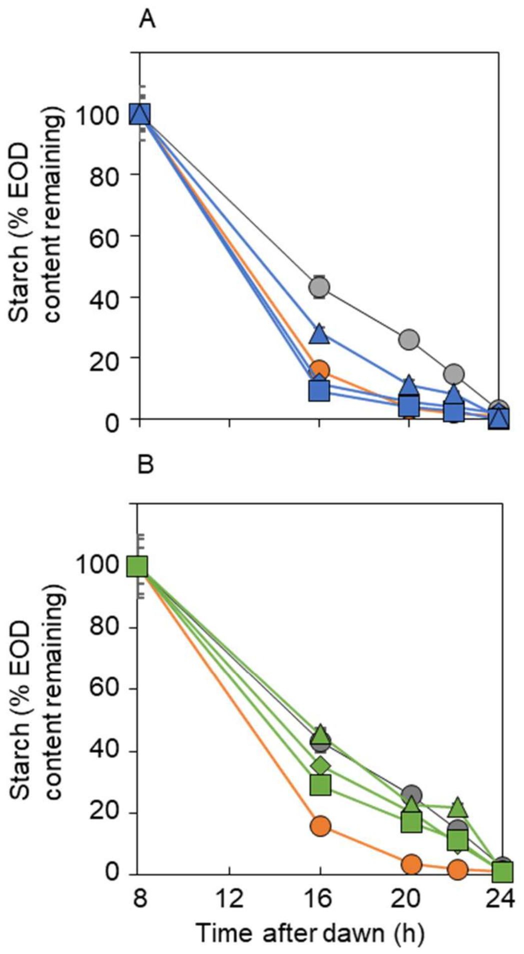

Supplemental Figure S4. Relative rates of starch degradation in *bam1-1* and *bam1-2D* plants and transgenic lines expressing BAM1(S132N). Data from Figure 4 are replotted as the % of end-of-day (EOD) starch remaining at intervals during the night, for an early night starting at 8 h after dawn. Grey symbols, *bam1-1*; orange symbols, *bam1-2D. (*A) Blue symbols represent transgenic lines B1.12.12 (triangle), B1.13.7 (square), and B1.2.21 (diamond). (B) Green symbols represent transgenic lines W1.9.8 (triangle), W1.1.7 (square), and W1.5.10 (diamond). Data are means of measurements on five individual rosettes. Error bars are SE: where not visible, they are smaller than the symbol.

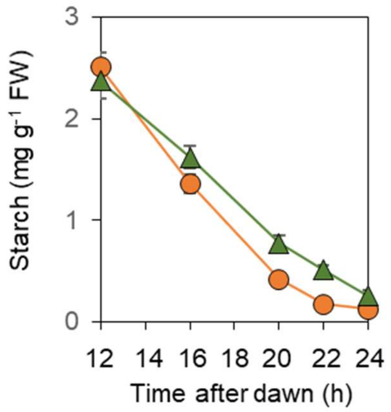

Supplemental Figure S5. Comparison of patterns of starch degradation in *bam1-2D* and a *bam1-2D lsf1 bam3* mutant. These measurements were made with a triple mutant selected independently of the triple mutant shown in Figure 6D, and using a separate batch of plants from that shown in Figure 6. Note that as for Figure 6D the rate of starch degradation in the triple mutant is linear and adjusted to the length of the night. Orange circle, *bam1-2D.* Green triangle, *bam1-2D lsf1 bam3*. Data are means of measurements on five individual rosettes. Error bars are SE: where not visible, they are smaller than the symbol

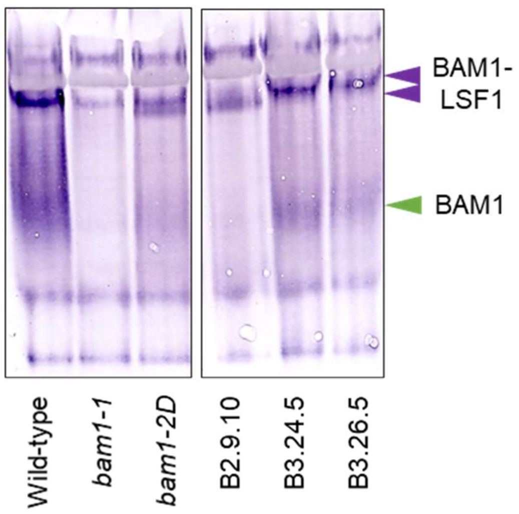

Figure S6. Immunoblot of a native gel of transformants expressing modified BAM1 proteins. The immunoblot is of the same extracts as those in Figure 5D, run on a separate gel.

Supplemental Table S1. Peptides recovered from BAM1 proteins. Highlighted regions are those from which peptides were recovered; numbers above the sequence show the designations used in the Table. The Table shows numbers of each designated peptide recovered from strips of gel containing the upper or the lower of the two bands attributable to BAM1 on immunoblots. S132 is shown in red.

```
            1                      2                                                    3                   4
MALNLSHQLGVLAGTPIKSGEMTDSSLLSISPPSARMMTPKAMNRNYKAHGTDPSPPMSPILGATRADLSVACKA
          5                                                6
FAVENGIGTIEEQRTYREGGIGGKKEGGGGVPVFVMMPLDSVTMGNTVNRRKAMKASLQALKSAGVEGIMIDV
                             7                                                                  8
WWGLVEKESPGTYNWGGYNELLELAKKLGLKVQAVMSFHQCGGNVGDSVTIPLPQWVVEEVDKDPDLAYTDQ
                    9                   10                           11                  12
WGRRNHEYPISLGADTLPVLKGRTPVQCYADFMRAFRDNFKHLLGETIVEIQVGMGPAGELRYPSYPEQEGTWK
          13                                 14
FPGIGAFQCYDKYSLSSLKAAAETYGKPEWGSTGPTDAGHYNNWPEDTQFFKKEGGGWNSEYGDFFLSWYSQM
    15                  16                 17                   18                     19                 20
LLDHGERILSSAKSIFENMGVKISVKIAGIHWHYGTRSHAPELTAGYYNTRFRDGYLPIAQMLARHNAIFNFTCIEMR
           21                        22                           23                 24                  25
DHEQPQDALCAPEKLVNQVALATLAAEVPLAGENALPRYDDYAHEQILKASALNLDQNNEGEPREMCAFTYLRM
          26                                                27
NPELFQADNWGKFVAFVKKMGEGRDSHRCREEVEREAEHFVHVTQPLVQEAAVALTH
```

Numbers of peptides recovered		Peptide designation																											Total
		1	2	3	4	5	6	7	8	9	10	11	12	13	14	15	16	17	18	19	20	21	22	23	24	25	26	27	
Wild-type	Upper band	4	3	1	4	0	2	1	0	9	0	4	1	0	0	0	1	1	4	1	2	6	6	5	4	2	3	7	71
bam1.2.21	Lower band	10	6	8	2	18	13	7	6	1	4	4	3	4	1	1	8	4	13	8	8	6	25	6	19	3	6	6	200

Genotype	After 8 h light (set to 1.00)	After 8 h dark, as a fraction of 8 h light value	Rate, as fraction h^{-1}
bam1-1	1.00 ± 0.06	0.43 ± 0.04	0.07
bam1-2D	1.00 ± 0.09	0.16 ± 0.01	0.11*
B2.2.21	1.00 ± 0.11	0.12 ± 0.02	0.11*
B2.13.7	1.00 ± 0.02	0.09 ± 0.01	0.11*
B2.12.12	1.00 ± 0.06	0.28 ± 0.02	0.09*
W2.10.5	1.00 ± 0.05	0.35 ± 0.05	0.08*
W2.9.8	1.00 ± 0.06	0.46 ± 0.02	0.07
W2.1.7	1.00 ± 0.1	0.29 ± 0.02	0.09*

Supplemental Table S2. Relative rates of starch degradation in the first 8 h of an early night in *bam1-1* and *bam1-2D* plants and transgenic lines expressing BAM1(S132N). Data from Figure S5 were used. Amount of starch after 8 h dark is expressed as a fraction of the amount after 8 h light. Data are means of measurements on five individual rosettes, ± SE. Rate of consumption as a fraction h^{-1} was estimated from the two sets of values. Asterisks indicate rates of consumption that were statistically significantly different from the rate in wild-type plants (Student's t, $P<0.05$).

Genotype	After 8 h light, set to 1.00	After 8h dark, as a fraction of 8 h light value
WT	1.00 ± 0.08	0.45 ± 0.04
bam1-2D	1.00 ± 0.13	0.10 ± 0.01*
amy3	1.00 ± 0.04	0.46 ± 0.04
bam4-1	1.00 ± 0.07	0.96 ± 0.06*
bam1-2D amy3	1.00 ± 0.18	0.14 ± 0.02*
bam1-2D bam4-1	1.00 ± 0.13	0.05 ± 0.02*

Supplemental Table S3. Starch consumption in the first 8 h of an early night in the wild-type and single and double mutant lines. Starch content (mg g^{-1} fresh weight) was measured after 8 h light and after 8 h dark. Amount after 8 h dark is expressed as a fraction of the amount after 8 h light. Values were calculated from means of measurements on five or six individual rosettes, ± SE. Asterisks indicate values after 8 h dark that were statistically significantly different from those in wild-type plants (Student's t, $P<0.05$).

Supplemental Table S4. Primers used in this study.

	AGI Number		Sequence 5’-3’	Further information
RT-qPCR primers:				
ACT2	AT3g18780	F	ACTTTCATCAGCCGTTTTGA	Reference gene
		R	ACGATTGGTTGAATATCATCAG	
UBQ10	AT4g0532	F	AGAACTCTTGCTGACTACAAT	Reference gene
		R	ATAGTTTTCCCAGTCAACGTC	
YLS8	AT5g08290	F	TTACTGTTTCGGTTGTTCTCCATTT	Reference gene
		R	CACTGAATCATGTTCGAAGCAAGT	
BAM1	AT3g23920	F	CCATTGTGGAAATCCAAGTG	*BAM1* expression in WT, *bam1-1, bam1-2*, *S132A* and *S132D*
		R	ACGAGTACTTATCATAGCACTG	
Site-directed mutagenesis primers:	AT3g23920		Base pair changes underlined	
1S132 to S132A		F	GCGATGAAAGCGGCGTTGCAAGCGTTGAA	Serine to alanine change
		R	GCCTTTCTTCGATTCACC	
S132 to S132D		F	GAAGAAAGGCGATGAAAGCGGATTTGCAAGCGTTGAAGAGTGC	Serine to aspartic acid change
		R	GCACTCTTCAACGCTTGCAAATCCGCTTTCATCGCCTTTCTTC	
Cloning primers	AT3g23920			
BAM1 & *bam1-2*		F	ATGGCGCTTAATTTATCGCAT	cDNA cloning
		R	CTAGTGAGTGAGAGCCACTGC	
BAM1 and bam1-2 promoters		F	ATTTGAAGAAAGCTTTAGTCTTGGG	2384bp of promoter sequence
		R	CATAATAAATTGAACCGGAATTG	
Genotyping primers:				Mutant KO confirmation
amy3-2	AT1g69830	F	TAACGTCACCCGGTAAATCG	SAIL_613 D12
		R	ATGAGACCTCCTGGCTCAAT	
bam1-1and bam1-2	AT3g23920	F	ACAAGGCTCACGGAACAGAT	*bam1-1* = SALK-039895
		R	AGACTCTCACGCAACAAAGAAA	
bam3-1	AT4G17090	F	GAACAAGTGGACCTCATGATG	CS92461
		R	TGAGAGTCTCCTCCCATGAC	
bam4-1	AT5g55700	F	TGGACTACCACCGGTAAAGC	SALK_037355
		R	GGCTGCCTTTGCAAGAATAG	
lsf1-1	AT3G01510	F	AGTAAGAGGAGCTCGCCGAC	SALK_053285
		R	TTCGAGAGCTCCTAAACCGG	
sex4-3	AT3G52180	F	AAGGAAATCCCCAAACATCC	SALK_102567
		R	TGTTCGTCCACCTTTGTCTG	
Left border primers:				
GABI_Kat			CCCATTTGGACGTGAATGTAGACAC	
SAIL			TAGCATCTGAATTTCATAACCAATCTCGATACAC	
SALK_LB1			GCGTGGACCGCTTGCTGCAACT	
Fine mapping primers				Sourced: http://amp.genomics.org.cn/
3-AB022217-2402F		F	ACCTGTTCAGTCTATGTTAC	
3-AB022217-2402R		R	GGGAATTATTAACATTATCA	
3-AP000731-3394F		F	GGTAAGCTTCAGGTCGTGCT	
3-AP000731-3394R		R	GTCAACACTTTGACCCGACA	
3-AP002461-5399F		F	GAAGCGGTGTGGCTTGAAC	
3-AP002461-5399R		R	GATGAACGCTTCAGGCCTAT	
3-AP000419-2846F		F	TTTGTTCGAGTTTAATACAT	
3-AP000419-2846R		R	ATGTGTCACTAGTCGTATTC	
3-AB025624-2876F		F	GCTTCTTCTCGTTGCGATCT	
3-AB025624-2876R		R	TTCAAATGGTGAAGCAGTAA	
3-AP001297-3676F		F	TCAAAAAGCTAAACGATACA	
3-AP001297-3676R		R	GGCGATTATAGAGAAACAGA	
3-AB028607-3997F		F	TTGCGTCTGGCAGATTGTTG	
3-AB028607-3997R		R	GGCCATATTTTGCTATGTTT	

3-AP001304-3123F	F	CCAAGGGAATCCAATGAAGC	
3-AP001304-3123R	R	CAATTCTGTATTATCGATGATGTG	
3-AB023045-3172F	F	GTAGCCCAAAGCCGTACAG	
3-AB023045-3172R	R	GAGATGCGTTTCACCTACAA	
3-AB026647-03900F	F	ATGGTAAGCCAAAAGTCATC	
3-AB026647-03900R	R	GTAATTCCAGACCTTGTAGA	
3-AB026655-3510F	F	CAATTCGATCGTCATTACAG	
3-AB026655-3510R	R	ATGTATCTCTTGGCTAAGTT	
3-AP002048-3789F	F	AGAGCTGGAGTCAAGTATC	
3-AP002048-3789R	R	CATCCAACTCATGACAAG	

Sequencing primers: AT3g23920

			BAM1 and *bam1-2*
BAM1_F1	F	GTCTTGGGGATTCATTTTGG	
Bam1_R1	R	TTCATGAAAGATCAATGATTCG	
BAM1_F2	F	TGGATTGCAACAACTCTTTCAT	
BAM1_R2	R	CGAAATCAAATCAATTTAATAACTCA	
BAM1_F3	F	TCGAAAACTACTTGTATTCATCAGAA	
BAM1_R3	R	TTTTCTTACAAGCAAACGGTGA	
BAM1_F4	F	GAACACTATCACCGTTTGCTTG	
BAM1_R4	R	TTCTTCAATGGTCCCGATTC	
BAM1_F6	F	ACGTGTACGGGAATTGATGA	
BAM1_R6	R	ATAGCACTGGAAGGCTCCAA	
BAM1_F7	F	ATTGGAGCCTTCCAGTGCTA	
BAM1_R7	R	TCTCTCGAGGTTCTCCCTCA	
BAM1_F8	F	GCACTTTGTGCACCAGAGAA	
BAM1_R8	R	GAAATGCTATTTATTTTGGCTTCA	
BAM1 PROMF1	F	TGAAGAAAGCTTTAGTCTTGGG	*BAM1* and *bam1-2* - promoters
BAM1 PROMR1	R	CATAATAAATTGAACCGGAATTG	
BAM1 PROMF2	F	CCCTCTAGTAGCCACATCTGC	
BAM1 PROMR2	R	CAAGATTGGATTATGGATTTC	
BAM1 PROMF3	F	CCACCACTCAACATTTTGTTTG	
BAM1PROMR3	R	CCTTCGATTCAAGTCAATAATC	
BAM1 PROMF4	F	CTTGTACATCATGTGATCTCACG	
BAM1 PROMR4	R	CTGACCTATGAATCGCTTTAC	
BAM1 PROMF5	F	CTGAGGCTAAAAGCGCGTCTGCTG	
BAM1 PROMR5	R	CATTTTTCTCTCTATACGCGAG	
